# Identification of characteristic aroma and bacteria related to aroma evolution during long-term storage of compressed white tea

**DOI:** 10.3389/fnut.2022.1092048

**Published:** 2022-12-19

**Authors:** Zhihui Wang, Zhihua Wang, Haomin Dai, Shaoling Wu, Bo Song, Fuming Lin, Yan Huang, Xingchen Lin, Weijiang Sun

**Affiliations:** ^1^College of Horticulture, Fujian Agriculture and Forestry University, Fuzhou, China; ^2^Ministerial and Provincial Joint Innovation Centre for Safety Production of Cross-Strait Crops, Fujian Agriculture and Forestry University, Fuzhou, China; ^3^Anxi College of Tea Science, Fujian Agriculture and Forestry University, Quanzhou, China; ^4^Fujian Ming Shan Tea Industry Co., Ltd., Fuding, China

**Keywords:** compressed white tea, storage, volatile compounds, GC-IMS, aroma, bacteria

## Abstract

Compressed white tea (CWT) is a reprocessed tea of white tea. Long-term storage has greatly changed its aroma characteristics, but the material basis and transformation mechanism of its unique aroma are still unclear. In this study, flavor wheel, headspace gas chromatography ion mobility spectroscopy, chemometrics, and microbiomics were applied to study the flavor evolution and important aroma components during long-term storage of CWT, and core functional bacteria were screened. During long-term storage, the aroma of CWT gradually changed from sweet, fruity and floral to stale flavor, woody and herbal. A total of 56 volatile organic compounds (VOCs) were identified, 54 of which were significantly differences during storage. The alcohols content was the highest during 1–5 years of storage, the esters content was the highest during 7–13 years of storage, and the aldehydes content was the highest during 16 years of storage. Twenty-nine VOCs were identified as important aroma components, which were significantly correlated with 6 aroma sub-attributes (*P* < 0.05). The functional prediction of bacterial community reminded that bacterial community could participate in the transformation of VOCs during storage of CWT. Twenty-four core functional bacteria were screened, which were significantly associated with 29 VOCs. Finally, 23 characteristic differential VOCs were excavated, which could be used to identify CWT in different storage years. Taken together, these findings provided new insights into the changes in aroma characteristics during storage of CWT and increased the understanding of the mechanism of characteristic aroma formation during storage.

## 1. Introduction

White tea is a kind of slightly fermented tea (1). After a long aging period, white tea can get unique aroma characteristic and then is named aged white tea. It is similar with aging process of Pu-erh tea and red wine (2, 3). However, the traditional white tea is coarse and loose, which is inconvenient for transportation and storage, so the compressed white tea (CWT) has been developed (4). CWT is a reprocessed tea product manufacture from white tea by blending, weighing, steaming, shaping, and drying. Its appearance effectively solved the problem of inconvenient transportation and storage of traditional white tea (5). Previous studies have expounded that the process of steaming and shaping further destroys the bud and leaf tissue of white tea, and has a far-reaching impact on the type and content of aroma components of CWT, making the stale flavor of CWT more show, and the characteristic aroma types such as jujube scent and herbal more prominent, while the traditional white tea retains more floral and fruity (6).

Aroma is an important factor that determines the quality of tea and affects the choice of consumers (7). When white tea is not stored, its aroma characteristics are floral, sweet, clean and refreshing, and the high proportion of alcohols and aldehydes is the basis of these aroma characteristics. Phenylethyl alcohol, γ-nonalactone, *trans*-β-ionone, *trans*-linalool oxide (furanoid), α-ionone and *cis*-3-hexenyl butyrate are considered to be the key aroma components of different types of unstored white tea (8). In the long-term storage process of white tea, the aroma will change with the extension of time (9). In the sensory, the aroma of white tea will gradually transform from clean and refreshing and floral to stale flavor, jujube scent or herbal (10). In the volatile organic compounds (VOCs), the content of alcohols decreases, hydrocarbons' increases, and aldehydes' increases first and then decreases; the aroma components of floral and fruity, such as linalool, linalool oxide, geraniol, methyl salicylate, phenylethanol, nerolidol, and citral, decreased, which make white tea clean and refreshing and floral gradually decrease or even disappear. A variety of unsaturated alkenes, mainly dihydrokiriactone, 2-methylnaphthalene, cedrene, and β-cedrene, are increased, resulting in the formation of stale flavor. Meanwhile, under the coordination of benzaldehyde, α-ionone, β-ionone, and geranyl acetone, the characteristics of white tea during storage, namely stale flavor with jujube scent, plumy aroma are formed (8, 11). The aroma of CWT is very different from that of traditional white tea (6). So far, the changes of aroma characteristics, important aroma components and the chemical basis of special aroma during storage of CWT are still unclear.

Headspace gas chromatography-ion mobility spectrometry (HS-GC-IMS) is an emerging technology, which can visualize VOCs without pretreatment and high sensitivity, and can better separate isomers and polymers in tea (12, 13). Nevertheless, how to match valuable information on the chemicals with sensory descriptors is still challenging. Recently, chemometrics and quantitative descriptive analysis (QDA) have been widely used to reveal the relationship between the chemical data from instrumental analysis and sensory analysis, and to recognize those chemical components that contribute significantly to food flavor (14, 15). However, there are few reports on the relationship between aroma characteristics and VOCs during storage of CWT. In addition, microorganisms may play a key role in the changes of tea metabolites during storage (3), which has been confirmed in Fu Brick tea, Liupao tea, and Pu-erh tea (16–18), and it is found that the diversity of bacteria increases gradually during the storage process of tea, mainly involved in the decomposition, transformation and degradation of small molecule compounds (17). Previous study have identified bacterial communities during storage of white tea and CWT, and performed functional prediction. It is found that bacteria can participate in 246 kyoto encyclopedia of genes and genomes (KEGG) metabolic pathways, including the metabolism of some VOCs (19, 20). Whereas, the relationship between the changes of VOCs and bacterial communities during storage of CWT are also still unknown.

Therefore, the aims of the present study were to (a) elucidate the dynamic changes in aroma characteristics and VOCs during storage of CWT, (b) elucidate the correlation between VOCs and aroma sub-attributes, and (c) identify important aroma components and core functional bacteria that contribute to the formation of characteristic aroma of CWT. This study is of significant importance for providing information in-depth to enhance the understanding of the mechanisms on aroma formation during storage of CWT.

## 2. Materials and methods

### 2.1. Experimental materials

**Figure 1:**
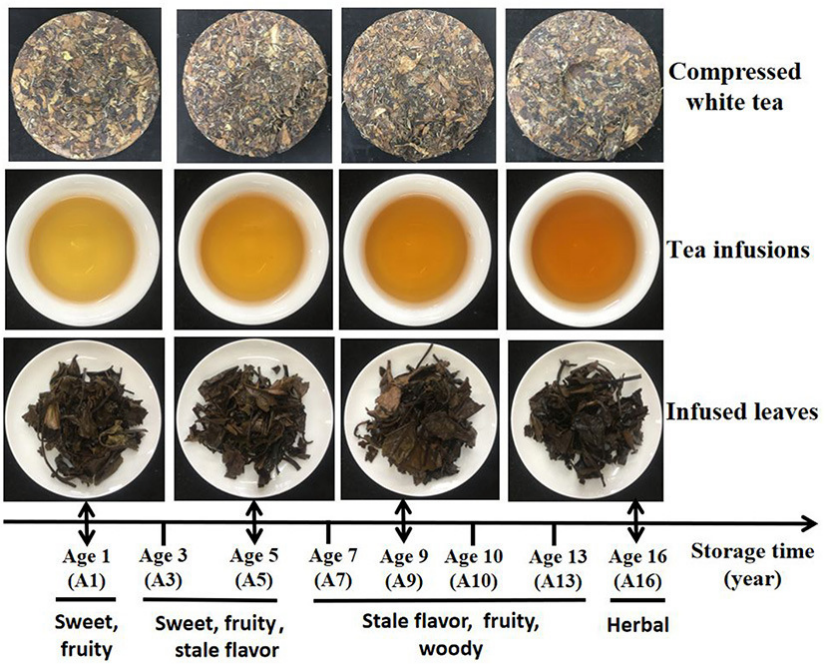
Photos of dry tea, tea infusions, and infused leaves during long-term storage of CWT.

Samples of CWT (round cake) were collected from Fujian Ruida Tea Industry Co., LTD., named A1, A3, A5, A7, A9, A10, A13, and A16, respectively. The last numbers in the nomenclature indicated the years of storage, which corresponded to the production years of 2018, 2016, 2014, 2012, 2010, 2009, 2006, and 2003. All samples were manufactured by Fujian Ruida Tea Industry Co., Ltd. in the spring with the same tea plant cultivar, plantation, and processing technology. The processing technology of CWT was that Shoumei white tea → weighing (weight: 350.0 ± 5.0 g) → steam softening (steam temperature: 110°C; time: 30 s) → shaping (round cake; pressure: 40 KN; time: 7 min) → drying (temperature: 50°C; time: 48 h; water content ≤ 8%). All samples were stored in the same environment warehouse (dry and ventilated, temperature ≤ 35°C, relative humidity ≤ 50%). The samples of different batches prepared within 1 month each spring were regarded as biological repeats, and each sample was collected with three biological repeats. The sample details are shown in Supplementary Table 1, and photos of dry tea, tea infusions and infused leaves of some CWT samples are shown in Figure 1.

### 2.2. Experimental method

#### 2.2.1. Sensory evaluation of the aroma of CWT

**Figure 2:**
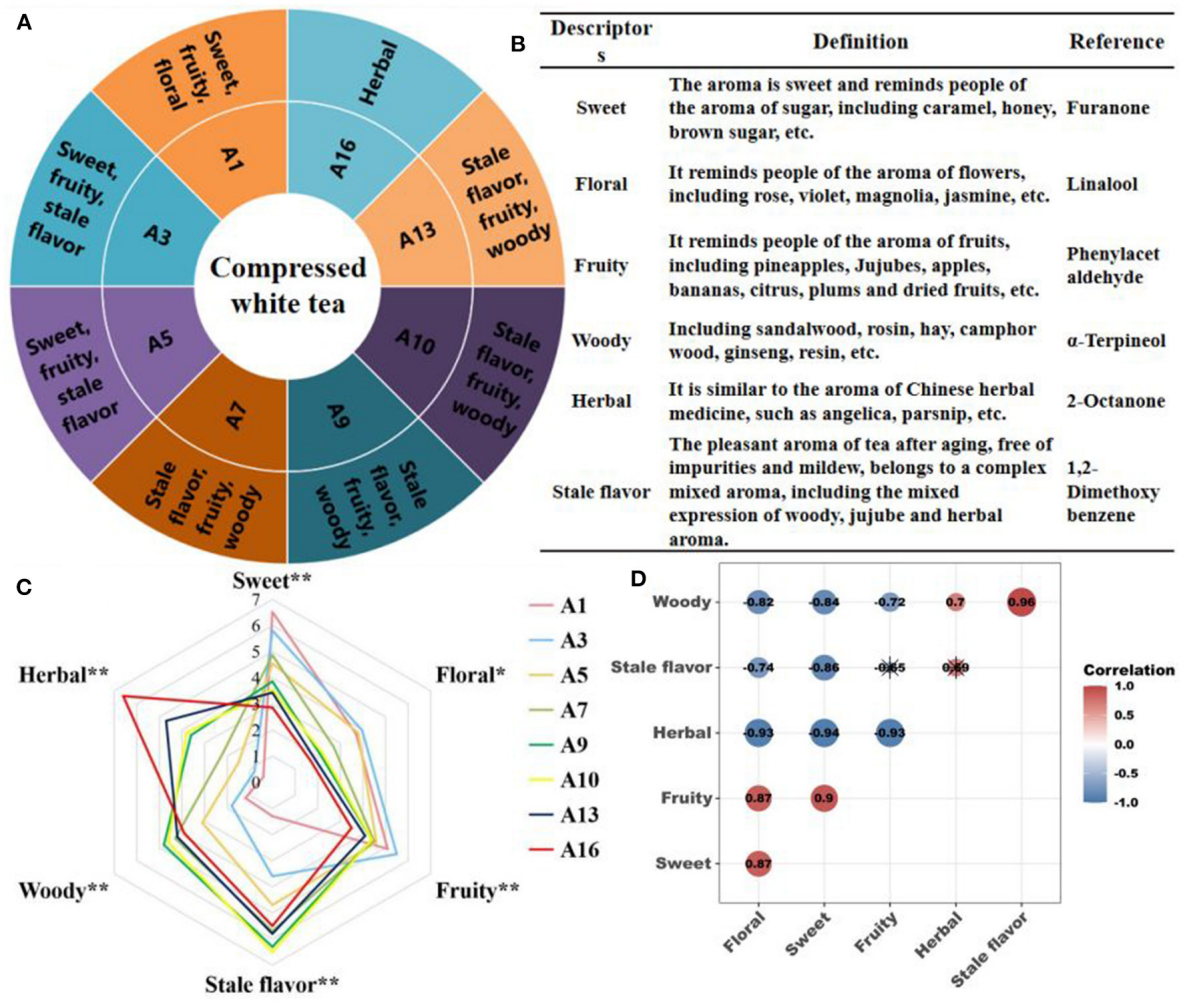
Sensory aroma characteristics of CWT. **(A)** Flavor wheel during storage of CWT. **(B)** Definition and reference of aroma descriptors. **(C)** Quantitative evaluation of aroma sub-attributes, *P < 0.05; **P < 0.01. **(D)** The intensity of the aroma sub-attribute correlation analysis, red and blue indicated positive and negative correlations, respectively. ^*^meant that the difference was not significant (*P* > 0.05).

Quantitative descriptive analysis (QDA) was performed by five well-trained panelists (two males and three females, aged 20–55 years) from the tea innovation team of Fujian Agriculture and Forestry University according to the Methodology for Sensory Evaluation of Tea (GB/T 23776-2018), with a little modification. Briefly, 3.0 g evenly mixed tea samples were weighed into a cylindrical cup of 150 mL, 150 mL boiling water was added, and soaked for 3 min. Then the tea infusion was poured into the evaluation bowl. After the initial evaluation of the aroma, the boiling water was added again, and the tea infusion was drained after 5 min for the second aroma evaluation. Aroma descriptors for each sample were recorded by each evaluator. After that, six descriptors with a usage rate of more than 80% were selected, namely sweet, floral, fruity, stale flavor, woody, and herbal. Descriptors were defined and their references were found based on published literature (Figure 2B) (21, 22). Finally, the tea samples were re-soaked according to the above method, and the 0–7 scale was used to evaluate the intensity of the six aroma sub-attributes of each sample, namely 0 = none, 4 = medium, and 7 = extremely. The aroma sub-attribute intensity value of each sample was the average value evaluated by the evaluation team. Each sample was evaluated in three biological replicates.

#### 2.2.2. Qualitative and quantitative analysis of the VOCs by HS-GC-IMS

VOCs identification analysis was performed using HS-GC-IMS (FlavorSpec, G.A.S., Dortmund, Germany) (23). Briefly, 0.1 g of the ground CWT sample was weighed and placed into a 20 mL headspace injection bottle. The headspace injection conditions were as follows: incubation at 70°C for 15 min with revolving speed at 500 *r*/min, injection needle temperature at 90°C, and injection volume at 500 μL. Three biological replicates were performed.

GC condition: the chromatographic column was FS-SE-54-CB-1 (15 mL × 0.68 mm), and the carrier gas was N_2_ (purity > 99.99%). The programmed flow rate was as follows: initially 2 mL/min holding for 10 min, linearly ramped up to 150 mL/min within 10 min, and then held for 30 min. IMS conditions: drift tube length and temperature were 98 mm and 45°C, drift gas flow rate was 150 mL/min. GC-IMS data were viewed through Laboratory Analytical Viewer (LAV), using the Reporter plug-in to directly compare 2D top view and 3D spectra, and the Gallery mapping plug-in to visualize fingerprint comparisons. Normal alkanes C9—C27 (Sigma Aldrich Corporation, Saint Louis, MO, USA) were served as external references to calculate the retention index (RI) of each compound. The calculation method of RI was referred to Mao et al. (24).

#### 2.2.3. Identification of bacterial communities

Previously, the bacterial communities of samples A1, A3, A7, A9, A10, and A16 during storage of CWT were identified (20). Primers were designed according to the V3 and V4 conserved regions of bacterial 16S rDNA for polymerase chain reaction amplification. Two-end sequencing of 2 × 250 bp was performed by NovaSeq PE250 sequencer (Kapa Biosciences, Woburn, MA, USA). Three biological replicates were performed.

### 2.3. Data analysis

One-way analysis of variance (ANOVA) with least significant difference (LSD) was performed using SPSS (version 19.0; Chicago, IL, USA). Bacterial involvement in metabolic pathways was predicted by PICRUSt2 function prediction analysis software (https://www.omicstudio.cn/tool). Principal component analysis (PCA), orthonormal partial least-squares discriminant analysis (OPLS-DA), orthonormal partial least-square variable import of project (OPLS-VIP), and two way orthogonal partial least-squares analysis (O2PLS) were performed using SIMCA (version 14.0, Umetrics, Umea, Sweden). Heat maps and correlation analysis were generated using Hiplot (https://hiplot-academic.com). The correlation network diagrams were generated using Cytoscape (version 3.9.1; Beijing, China).

## 3. Results and Discussion

### 3.1. Flavor evolution during storage of CWT

QDA was used to construct the flavor wheel of CWT during storage (Figure 2A) and formed its aroma description system (Figure 2B). During the long-term storage of CWT, the characteristics of six typical aroma sub-attributes were happened significant (*P* < 0.05) or extremely significant changes (*P* < 0.01) (Figure 2C). The intensity of sweet, floral and fruity was significantly decreased with increasing storage years, while that of stale flavor, woody, and herbal emerged a significant increasing trend (Figure 2C). The aroma intensity of CWT in the first year of storage was dominated by sweet, fruity and floral, which were 6.5, 5.1, and 3.7, respectively. During 3–5 years of storage, the intensity of stale flavor gradually increased, and that of floral was gradually covered, with sweet, stale flavor and fruity as the dominant flavor. During 7–13 years of storage, the intensity of woody increased significantly, while that of sweet and floral decreased significantly. In this period, stale flavor, fruity and woody were the dominant flavor. After 16 years of storage, the intensity of herbal increased significantly and became the dominant flavor, with an aroma intensity of 6.6 (Figures 2A,C). In general, the change course of CWT aroma during long-term storage was as follows: sweet, fruity and floral → sweet, fruity and stale flavor → stale flavor, fruity and woody → herbal. The change trend was similar to the change of white tea aroma during storage (10). The difference was that CWT had no clean and refreshing at the beginning of the storage, and the floral was weaker. After 3 years of storage, CWT had shown obvious stale flavor, and the aging speed was faster than that of white tea.

There was a complex correlation between the six aroma sub-attributes (*P* < 0.05) (Figure 2D). Sweet, fruity and floral were significantly positively correlated with each other, and these three sub-attributes were significantly negatively correlated with woody and herbal, respectively, indicating that the aroma compounds contributing to the three sub-attributes might be similar. There was a significant positive correlation between woody and both stale flavor and herbal. There was a positive correlation between herbal and stale flavor, but the correlation did not reach a significant level (*P* > 0.05). Some studies have suggested that the biochemical basis of stale flavor in tea was aromatic compounds with pleasant woody and herbal (6, 25). This was similar to the conclusion of this study.

### 3.2. Changes of VOCs during storage of CWT

**Figure 3:**
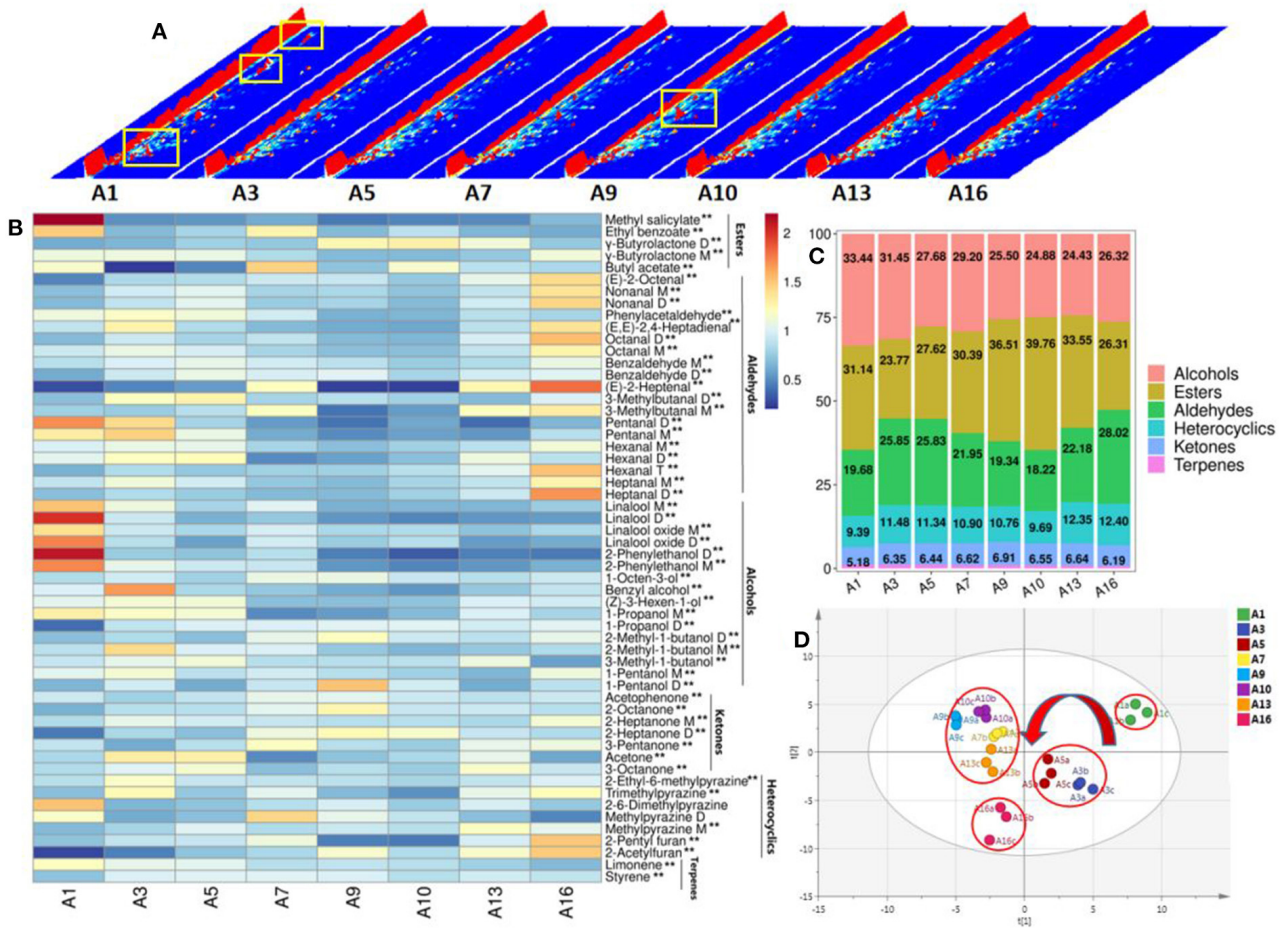
VOCs change during storage of CWT. **(A)** Total atlas of VOCs. **(B)** Heat map of VOCs change. Low levels in blue and high levels in red. **(*P* < 0.01). **(C)** The column diagram of the proportion of VOCs species. **(D)** Principal component analysis of VOCs. *R*^2^*X* = 0.98, *Q*^2^ = 0.816.

The general chromatogram of HS-GC-IMS showed that the relative abundance of VOCs in CWT during storage, and some VOCs increased and some decreased (yellow box) (Figure 3A). A total of 56 VOCs were identified, including 5 esters, 19 aldehydes, 16 alcohols, 7 ketones, 7 heterocycles, and 2 terpenes (Figure 3B). CAS, molecular formula, RI and relative content of all VOCs are shown in Supplementary Table 2. The most abundant VOCs in CWT were alcohols, esters and aldehydes. The dominant volatile components during long-term storage were constantly changing (Figure 3B). The alcohols content was the highest during 1–5 years of storage, the esters content was the highest during 7–13 years of storage, and the aldehydes content was the highest during 16 years of storage. The content of alcohols decreased gradually during long-term storage, ranging from 24.43 to 33.44%, and reached the highest level after 1 year of storage. During storage, esters first decreased, then increased, and then decreased, with the content ranging from 23.77 to 39.76%. The content was the highest after 10 years of storage. The variation trend of aldehydes was “N”, and the content of aldehydes ranged from 18.22–28.02%, with the highest content after 16 years of storage. The contents of heterocycles, ketones and terpenes were relatively low, and heterocycles' and ketones' gradually increased, while terpenes' gradually decreased. Previous study has claimed that the contents of alcohols, aldehydes and hydrocarbons in aged white tea are the highest (11). During storage of white tea, alcohols decrease continuously, hydrocarbons increase, and aldehydes increase first and then decrease (11). There were some differences with the conclusions of this study, which might be the difference between white tea and CWT. The content of esters in CWT was higher and further increased during storage, while aldehydes increased in a fluctuating manner. The increase of some esters and aldehydes might be caused by microbial metabolism, while the decreased of alcohols might be due to their own volatilization. Previous studies have shown that some alcohols gradually evaporate during tea storage (15). Some esters and aldehydes can be biosynthesized by microbial enzymes using precursors such as amino acids and fatty acids during tea fermentation (26).

The most abundant VOCs in CWT were γ-butyrolactone D (dimer) (13.79–29.44%), linalool M (3.99–9.54%), ethyl benzoate (3.89–9.51%), 1-propanol D (3.32–8.55%), linalool oxide M (3.27–6.39%), 2-ethyl-6-methylpyrazine (4.18–6.18%), and γ-butyrolactone M (monomer) (2.27–3.70%) (Supplementary Table 2). Linalool and its oxides have always been considered as VOCs with high content in white tea (27). This view was also similar in CWT. The high content of γ-butyrolactone in CWT was identified for the first time. γ-Lactone is the most common type of lactone, but less are identified by ordinary GC-MS (28). Xiao et al. (29) identified γ-butyrolactone in Fu brick tea from different producing areas by using GC-IMS, but failed to do so by using GC-MS. Therefore, it was speculated that GC-IMS have unique advantages in identifying γ-butyrolactone and its dimer. Zhu et al. (9) performed targeted identification of part of lactones in white tea with different storage times (unidentified γ-butyrolactone) through enantioselective gas sterics-mass spectrometry (Es-GC-MS), it was found that some lactones accumulated with the increase of white tea storage time. In the study of wine, the content of γ-lactone is also found to be related to the aging of wine (30, 31). Therefore, long-term storage of CWT might contribute to γ-butyrolactone accumulation. Among the 56 identified VOCs, 54 had significant differences in accumulation during storage (*P* < 0.01). Heat map analysis certified that γ-butyrolactone D increased significantly, γ-butyrolactone M rised first and then decreases, and some alcohols such as linalool M and linalool oxide M decreased significantly. Methyl salicylate and ethyl benzoate decreased significantly. Heterocycles such as 2-ethyl-6-methylpyrazine and methylpyrazine M increased significantly. Aldehyde such as benzaldehyde D and 3-methylbutanal D increased significantly (Figure 3C). The differential accumulation of these VOCs changed the flavor of CWT during storage, but not all VOCs contributed to the aroma. Therefore, chemometrics should be further combined to find the important aroma components.

PCA showed the similarities and differences of samples in CWT during storage. In terms of differences, there was no coincidence among samples, indicating that VOCs were different among samples (Figure 3D), which was consistent with the conclusion of heat map analysis (Figure 3B). From the perspective of similarity, the whole storage process was divided into four stages: stage 1: A1; stage 2: A3–A5; stage 3: A7–A13, stage 4: A16 (the closer the distance, the more similar) (Figure 3D). The four stages of the storage process divided by PCA were consistent with the changes of the sensory attributes of aroma mentioned above (Figure 2A). Based on the above analysis, it could be reasonably inferred that the flavor change of CWT during storage was due to the change of VOCs content, which might be related to the volatilization of compounds and the metabolism of microorganisms during storage.

### 3.3. Characteristic differential VOCs of CWT in different storage years

**Figure 4:**
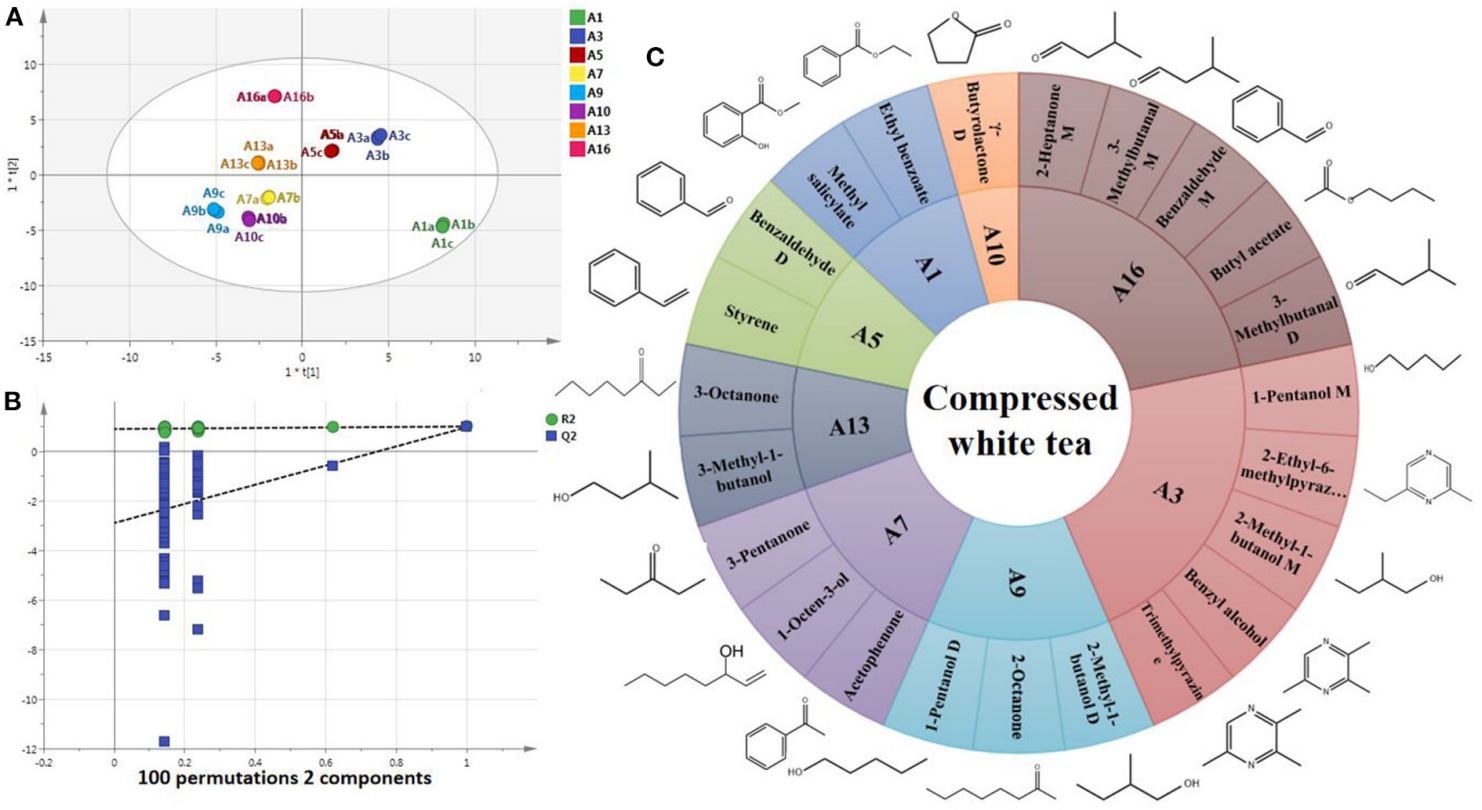
Characteristic differential VOCs of CWT in different storage years. **(A)** OPLS-DA model. *R*^2^*X* = 0.999, *R*^2^*Y* = 0.998, *Q*^2^ = 0.862. **(B)** Cross validation of OPLS-DA model. **(C)** Rising sun chart and molecular formula of characteristic differential VOCs.

PCA confirmed differences between CWT samples from different storage years. In order to screen the differential VOCs of CWT in different years, OPLS-DA modeling analysis was performed. Samples were effectively distinguished in the score chart (Figure 4A), and cross-validation informed that the model was not overfitted (green points were below blue points) (Figure 4B). With VIP > 1, *P* < 0.05 as the criterion (32), 23 differential VOCs were screened. The screening of characteristic differential VOCs is based on the variable variation trend of differential VOCs in the heat map (Figure 3B). This method has been used accurately in the screening of characteristic differential metabolites of tea from different grades and different regions (33, 34). The differential VOCs with high content in the sample of this storage year but low content in other storage years were considered as the characteristic differential VOCs of CWT of this year. Therefore, the characteristic differential VOCs were screened as follows: 2 in A1, 5 in A3, 2 in A5, 3 in A7, 3 in A9, 1 in A10, 2 in A13, and 5 in A16 (Figure 4C). These characteristic differential VOCs were differential VOCs accumulated specifically by CWT in a certain year, which might contribute significantly to the formation of its unique flavor, and could also be used to trace and identify the origin of CWT in different storage years. However, the reasons for the unique accumulation of these VOCs in different storage years needed to be further studied.

### 3.4. Screening of important aroma components during storage of CWT

In order to find essential variables that are the most influential on the sensory attributes of tea aroma, especially when multicollinearity exists among variables, a OPLS-VIP is conducted to explain sensory attribute intensities with volatile component content as the *X* variable and sensory attribute intensity as the *Y* variable, and the VIP value of each *X* variable is calculated (24). The variables whose VIP values are >1 are considered to be the important aromatic compounds (21).

**Figure 5:**
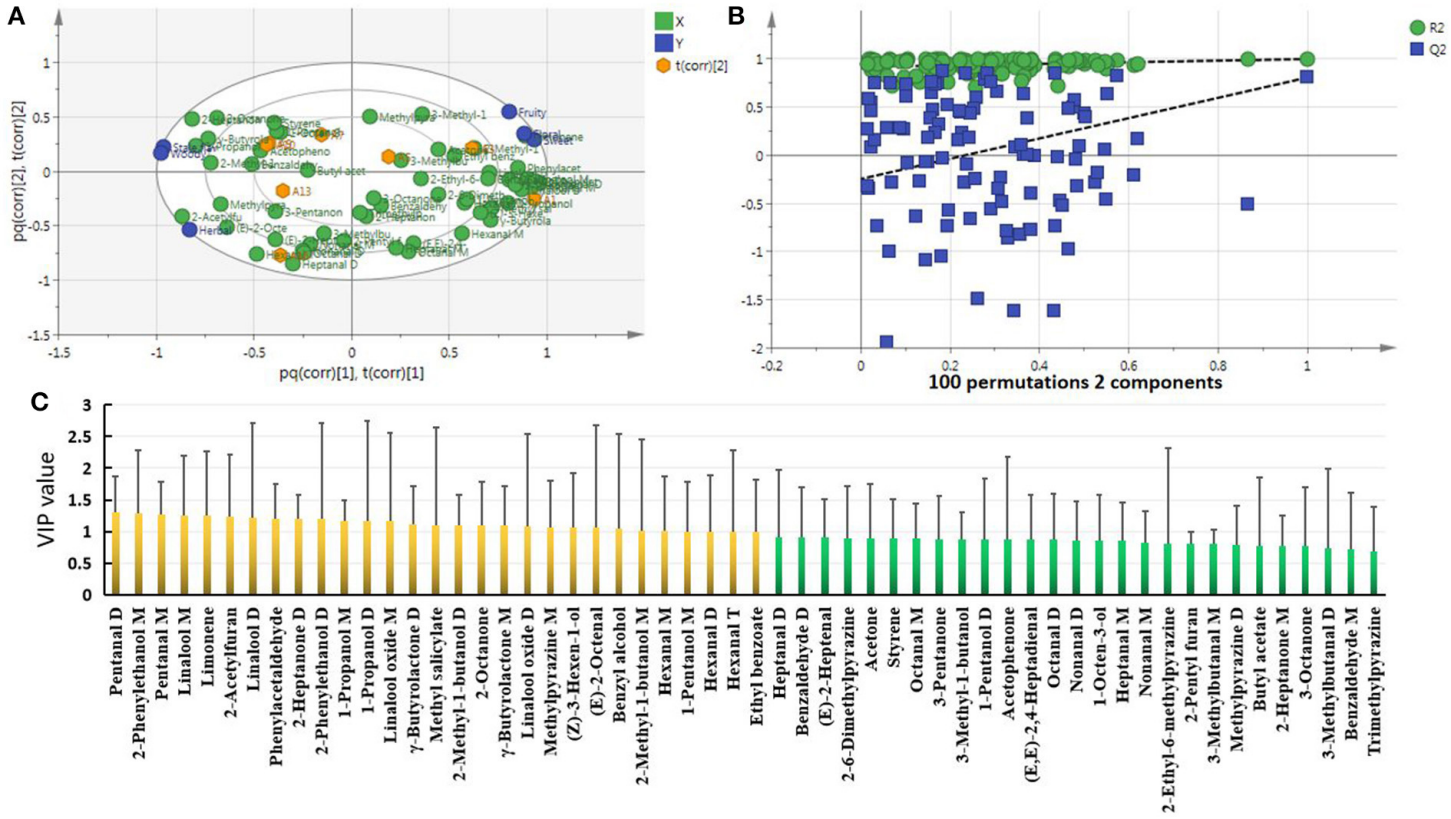
Screening of important aroma components during CWT storage. **(A)** OPLS-VIP model. *R*^2^*X* = 0.973, *R*^2^*Y* = 0.976, *Q*^2^ = 0.616. **(B)** Cross validation of OPLS-VIP model. **(C)** VIP value of VOCs.

The OPLS-VIP model had a high degree of interpretation (*R*^2^*X* = 0.973, *R*^2^*Y* = 0.976) (Figure 5A), and the closer the variables were, the greater the correlation was. Figure 5A showed that floral, fruity and sweet fragrance were the main aroma characteristics of A1, A3, and A5 (closer to each other), and stale flavor, woody and herbal were the main aroma characteristics of A7, A9, A10, A13, and A16 (closer to each other), which was consistent with the results of the above sensory analysis. Cross-validation informed that the model was not overfitted (Figure 5B). According to the principle of VIP value > 1.0, 29 volatile components were identified as aroma components that had important contributions to CWT sensory aroma (Figure 5C, yellow column). The aroma characteristics of these VOCs are relatively diversified, mainly including sweet, fruity, woody, caramel and fatty (Supplementary Table 2) (35, 36). These important aroma components acted as the aroma skeleton of CWT and interacted with other aroma components to form the unique aroma characteristics of CWT in different storage years. Linalool, linalool oxide, methyl salicylate, benzyl alcohol, 2-phenylethanol, and phenylacetaldehyde were closer to A1 and A3 (Figure 5A), and their aroma characteristics were fruit, sweet and floral (Supplementary Table 2) (35, 36). Many studies have verified that these VOCs are important aroma substances in fresh white tea (6, 11). In this study, chemometric methods were adopted to more accurately confirm the dominant contribution of these VOCs at the initial stage of CWT storage. γ-Butyrolactone D was closer to A7, A9, A10, A13, and A16 (Figure 5A). The thresholds of lactone compounds are extremely low, most of which have floral, fruity, milk flavor, and coconut, and are the important aroma substances of wine and fruit (9). There are also a variety of lactones in tea, which are mainly derived from γ(δ)-hydroxy carboxylic acid intramolecular esterification or carotene degradation during processing and storage. γ-Valerolactone, γ-heptanolactone, and γ(δ)-decanolactone have important contributions to tea quality (28). In this study, γ-butyrolactone was found to be a important aroma component in CWT storage process for the first time. It was likely that this is an important reason for the unique aroma of CWT.

### 3.5. Contribution of important aroma components to aroma sub-attributes

**Figure 6:**
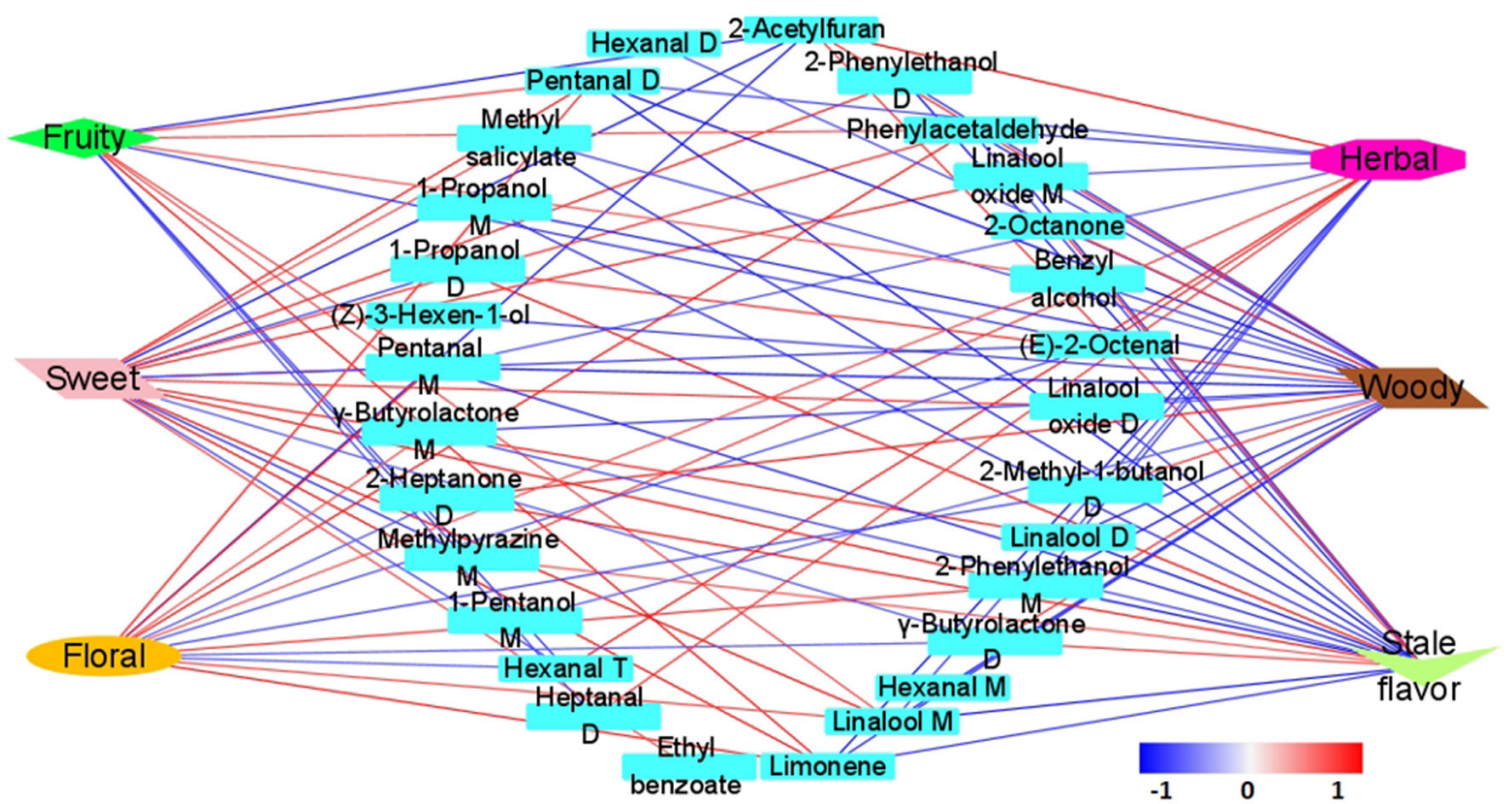
Correlation network diagram of aroma sub-attributes and key aroma components. Blue represented a significant negative correlation, red represented a significant positive correlation (*P* < 0.05), and the darker the color, the greater the correlation.

The correlation coefficient was used to determine the unique contribution of 29 important aroma components to six aroma sub-attributes. The 29 important aroma components were significantly correlated with six aroma sub-attributes (*P* < 0.05) (Figure 6). The correlation coefficient is shown in Supplementary Table 3, and the visual network diagram is shown in Figure 6.

Twelve VOCs were positively correlated with sweet, and six VOCs were negatively correlated with sweet. Six VOCs were positively correlated with fruity, and five VOCs were negatively correlated with fruity. There were nine VOCs positively correlated with floral, and six VOCs negatively correlated with floral (*P* < 0.05) (Figure 6). Linalool M, phenylacetaldehyde, pentanal D, pentanal M, and limonene were positively correlated with sweet, floral, and fruity. The aroma characteristics of these components are sweet, floral, fruit, citrus, lemon and cherry like (Supplementary Table 2) (35, 36). So they had a positive contribution to these aroma sub-attributes. The contents of these VOCs decreased significantly during storage (Figure 3B). (E)-2-Octenal and hexanal T were significantly negatively correlated with these three aroma sub-attributes, and the aroma characteristics of these two components are herbal, fatty and grass fragment (Supplementary Table 2) (35, 36). Therefore, it had a negative effect on sweet, floral and fruity. The contents of these two aroma components increased significantly during storage (Figure 3B). Sweet, floral and fruity had similar contribution components, which also verified the correlation analysis results among the above three (Figure 2B). The VOCs that had significant contribution to the aroma intensity of sweet, floral, and fruity also different. The VOCs with significant positive correlation with sweet also included methyl salicylate, ethyl benzoate, linalool D, linalool oxide M and linalool oxide D. The aroma characteristics of these components are mainly sweet, floral, fruity, honey-like and fresh (Supplementary Table 2). The VOCs with significant negative correlation with sweet were γ-butyrolactone D, 1-propanol D and methylpyrazine M, the aroma characteristics of these VOCs are caramel, cream, milk, fatty, ethanol-like, roasty, nutty and earthy (Supplementary Table 2). This hinted that the VOCs with caramel, fatty, roasty, and earthy were possible to reduce the sweet intensity of CWT. Benzyl alcohol (apple-like) had a significant positive correlation with fruity. γ-butyrolactone M (caramel) and 1-propanol M (pineapple) were significantly positively correlated with floral. The contents of these components of CWT decreased gradually during storage.

Six VOCs were positively correlated with stale flavor, and thirteen VOCs were negatively correlated with stale flavor. Six VOCs were positively correlated with woody, and eighteen VOCs were negatively correlated with woody. There were five VOCs positively correlated with herbal, and eight VOCs negatively correlated with herbal (*P* < 0.05) (Figure 6). γ-Butyrolactone D, 1-propanol D, and 2-heptanone D were positively correlated to the stale flavor and woody. The aroma characteristics of these three VOCs are carmel, cream, milk, fatty, herbal, and banana-like (Supplementary Table 2) (35, 36). The contents of these three VOCs increased gradually during storage. γ-Butyrolactone M, phenylacetaldehyde, pentanal D, pentanal M, linalool M, linalool D, linalool oxide M, linalool oxide D, 1-propanol M, and limonene were negatively correlated with stale flavor and herbel. Their aroma characteristics are sweet, floral and fruity (Supplementary Table 2). The content of these VOCs decreased gradually during storage. Different with woody, methylpyrazine M showed significant positive correlations with the stale flavor. The aroma characteristic of methylpyrazine M is roasty, nutty, and earthy (Supplementary Table 2). This suggested that VOCs with roasty, earthy, nutty, and herbal might affect the strength of stale flavor. It has obvious stale flavor in the aged white tea, Pu-erh tea, and Liupao tea (19). However, there were differences in the VOCs identified to contribute to stale flavor in different tea types. In Pu-erh tea, which is methoxybenzenes (37); in Liupao tea, which is α-cedarol, β-linalool, dihydrokiristone, α-terpineol„ and β-ionone (38); in aged white tea, which is cedarol, α-cedarene, and β-cedarene (6). Deng et al. (25) believed that the material basis of stale flavor in tea was mainly aromatic substances with pleasant woody and herbal. The results of this study were similar to this conclusion. Therefore, it was hypothesized that the reason for the difference in VOCs contributing to stale flavor among different tea types was the result of the interaction between these components. Different from stale flavor and wood, the VOCs that were significantly positively correlated with herbal were mainly (E)-2-octenal, (E)-2-heptenal, hexanal T, heptanal D, and methylpyrazine M. The aroma characteristics of these VOCs are herbal, fatty, grassy, stale flavor, fruity and cilantro (Supplementary Table 2). The contents of these VOCs gradually increased during storage. The seven VOCs negatively correlated with the herbal are mainly floral, fruity, and sweet. Their contents decreased gradually during storage of CWT.

In general, during the storage process of CWT, the important VOCs that were positively correlated with sweet, fruity, and floral aroma gradually decreased, while the components that were negatively correlated with them gradually increased. However, the important VOCs that were positively correlated with stale flavor, woody, and herbal gradually increased, while those that were negatively correlated with them gradually decreased. This was the main reason why the aroma of the CWT changed from sweet, floral, and fruity to stale flavor, woody, and herbal during storage.

### 3.6. Functional prediction of bacterial community

**Figure 7:**
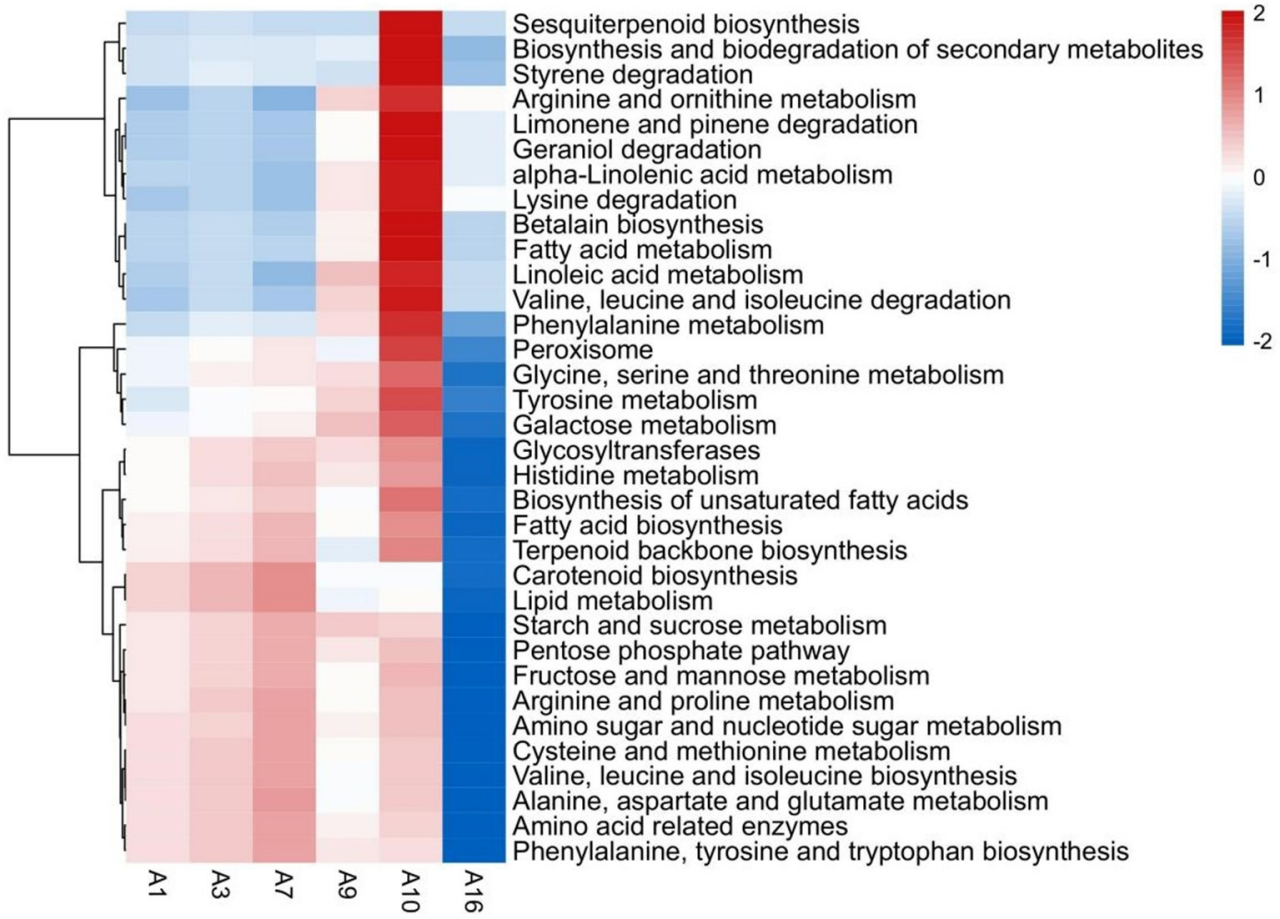
Intensity thermogram of KEGG pathway related to bacterial involvement in VOCs metabolism. Red indicated high intensity, while blue indicated low intensity.

Previous studies have found that microorganisms play an important role in the transformation of tea aroma during storage, among which bacteria may play a key role (17). Combined with the previous bacterial community data of CWT in different storage years (Supplementary Table 4) (20), PICRUSt2 was used to predict and analyze the function of bacteria, and acquire the information that bacteria in CWT storage process could participate in 264 KEGG metabolic pathways. Among them, 34 pathways were related to the metabolic transformation of VOCs and aroma precursors (Figure 7). The formation of tea aroma is divided into four ways: degradation of carotenoids, oxidative degradation of lipids, maillard reaction of sugars and amino acids, and hydrolysis of glycosides (25). The 34 metabolic pathways screened by us were all related to the four pathways of tea aroma formation, mainly including amino acid and carbohydrate metabolism, fatty acid metabolism, glycosyltransferase synthesis, peroxidase synthesis, terpene degradation and secondary metabolite biosynthesis, and biodegradation. The styrene degradation, α-linolenic acid metabolism, limonene and pinene degradation participated by bacteria first increased and then decreased during storage of CWT (Figure 7). The styrene content first increased and then decreased during storage, and the limonene content gradually decreased during storage (Figure 3B). α-Linolenic acid metabolism could synthesize (Z)-3-hexen-1-ol (39), and that content decreased first and then increased during storage (Figure 3B). Moreover, bacteria could participate in the metabolism of amino acid related enzymes, glycosyltransferases and peroxisome (Figure 7). These enzymes can be involved in the synthesis or degradation of tea aroma (39). Previous studies have shown that bacteria can secrete extracellular enzymes during tea storage, thereby affecting the quality of tea (17). This study also verified this conclusion through the prediction of bacterial function. In conclusion, it was reasonable to speculate that bacteria could participate in the transformation of VOCs during storage of CWT and play an important role, but not all bacteria did this. Therefore, the core functional bacteria affecting VOCs were further screened.

### 3.7. Screening of core functional bacteria affecting the change of VOCs

**Figure 8:**
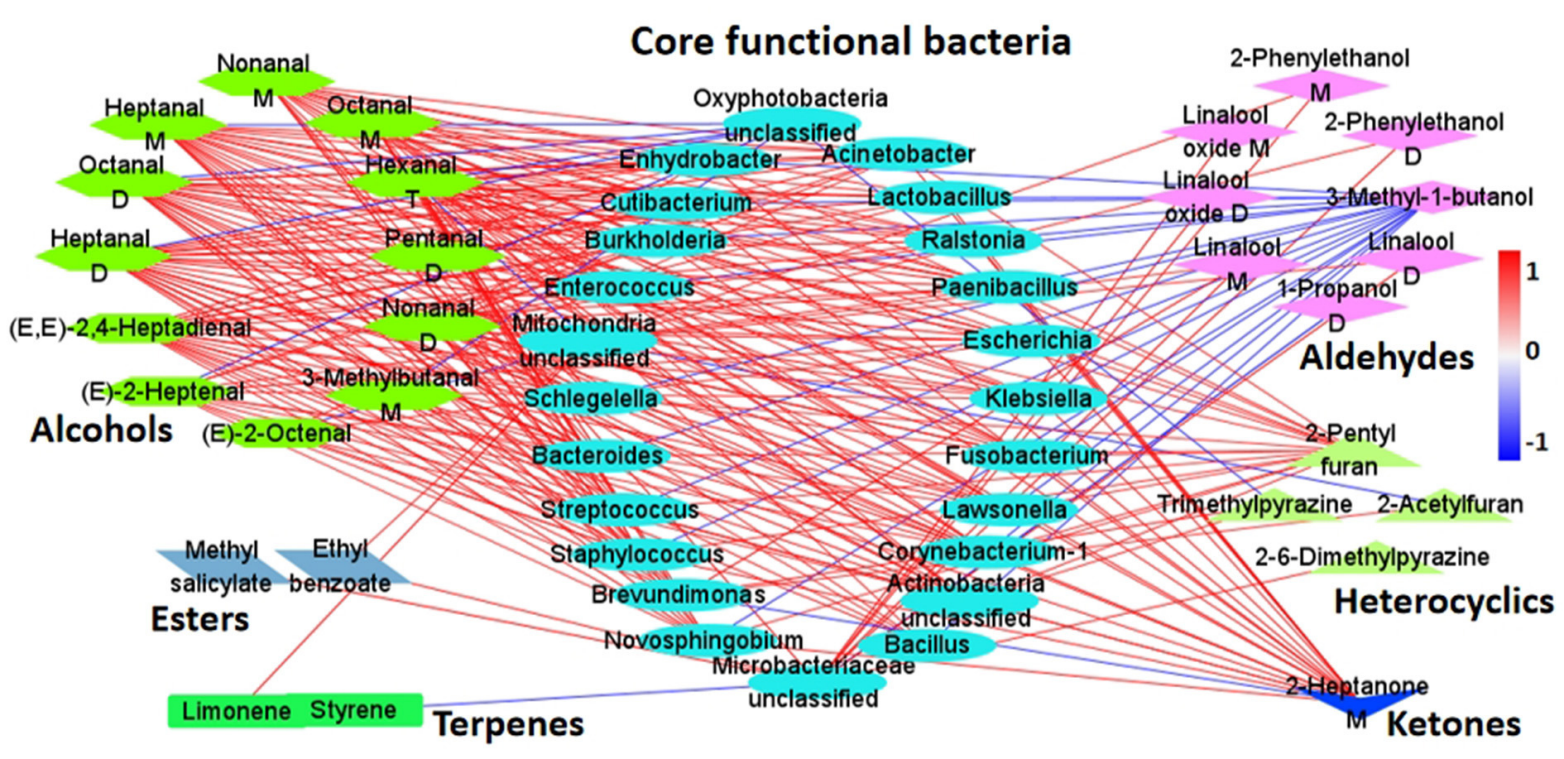
Correlation between core functional bacteria and VOCs. The red line indicated a significant positive correlation, and the blue line indicated a significant negative correlation, and the darker the color, the greater the correlation (*P* < 0.05).

Combined with the identified VOCs content and bacterial abundance data, an O2PLS model was established to study the association between bacteria and VOCs during storage of CWT. The O2PLS model had a high degree of interpretation (*R*^2^*X* = 0.791, *R*^2^*Y* = 0.797) (Supplementary Figure 1). Cross-validation showed no overfitting (Supplementary Figure 2). According to the principle of VIP value > 1.0, 116 bacterial genera were confirmed to have important influence on VOCs (Supplementary Table 5). Furthermore, three conditions were considered to identify the core functional bacteria that had an important contribution to the VOCs during storage of CWT: (a) VIP value > 1.0; (b) correlation coefficient | *r* | > 0.8 and *P* < 0.05; (c) the relative abundance of bacterial genera must be >1.0% (15, 16, 40, 41). Based on three criteria, 24 bacterial genera were identified as core functional bacteria. Core functional bacteria were significantly correlated with 29 VOCs (17 were important aroma components), including 12 aldehydes, 8 alcohols, 2 esters, 4 heterocycles, 2 terpenes, and 1 ketone (Figure 8). Correlation coefficients are shown in Supplementary Table 6. It could be seen from the network diagram that the core functional bacteria were the greatest correlation with 12 aldehydes, and these aldehydes tended to increase in the storage process (Figure 3B). The aroma precursors of hexanal, heptanal, and pentanal are lipids, and the aroma precursors of 3-methylbutanal M are amino acids (12, 42). Among the core functional bacteria, *Bacillus, Brevundimonas, Lactobacillus*, and *Enterococcus* can produce a large number of various types of extracellular enzymes (43, 44). In Liupao tea, Fu brick tea, and Pu-erh tea, microorganisms have been found to affect the aroma quality of tea by secreting extracellular enzymes (15, 16, 29). Therefore, it was speculated that these core functional bacteria promoted the degradation of amino acids and fatty acids by secreting extracellular enzymes, thus making the rise of these aldehydes aroma components. Similar to aldehydes were ketones. The precursor substances of 2-heptanone M are carotenoids (42). The content of alcohols such as linalool, 1-propanol and 2-phenylethanol decreased gradually during storage. Previous studies deem that the decline of alcohols during tea storage is related to its volatilization (15). This study found that *Mitochondria unclassified* and *Microbiaceae unclassified* were significantly correlated with content change of alcohols. Moreover, these two core functional bacteria were also significantly correlated with the content change of esters (methyl salicylate and ethyl benzoate) and terpenes (limonene). At present, there was no report on the functions of these two bacteria. So it was not clear how these two bacteria affected the decline of alcohols, esters and terpenes. *Oxyphotobacteria unclassified* was the dominant bacteria in CWT with the abundance range of 9.02–93.14%, which increased first and then decreased during storage (Supplementary Table 4). It was positively correlated with the content changes of most alcohols, and negatively correlated with the content changes of many aldehydes and heterocycles. *Oxyphotobacteria unclassified* exists in a large number of food fermentation processes, and its abundance gradually decreases with the increase of the degree of fermentation (45). However, the main function of this bacterium was still unclear, and further experiments needed to be designed and studied. These findings collectively indicated that core functional bacteria made a key contribution to the characteristic aroma formation during storage of CWT.

## 4. Conclusion

This study was the first report on the flavor evolution and dynamic changes of VOCs of CWT during long-term storage (1–16 years), and attempted to correlate them with bacterial communities. It was of great significance for us to further understand the flavor transformation and the formation mechanism of characteristic aroma of CWT during storage. The flavor wheel and aroma description system for CWT during storage was constructed, and 29 VOCs were identified as important aroma components, among which the unique contribution of γ-butyrolactone to the aroma of CWT was first discovered. According to the flavor wheel and VOCs, the storage process of CWT was divided into four stages. The change process of aroma was sweet, fruity, and floral (1 year) → sweet, fruity, and stale flavor (3–5 years) → stale flavor, fruity, and woody (7–13 years) → herbal (16 years). The reason for the change of flavor was that the important aroma components which were positively correlated with sweet, fruity and floral gradually decrease, while the components that were positively correlated with stale flavor, woody and herbal gradually increased. The functional prediction of bacterial communities revealed that bacteria participated in 34 metabolic pathways related to VOCs transformation, and 24 bacterial genera were identified as core functional bacteria. In addition, 23 characteristic differential VOCs of CWT in different storage years were screened out, which could be used to distinguish CWT in different years. Since the standard of all VOCs could not be obtained, the absolute quantification of these VOCs was not carried out, and the aroma recombination or omission experiment was not used for verification. Therefore, only chemometrics was used in this study to screen VOCs that had an important contribution to the sensory aroma sub-attributes of CWT. Further studies should use molecular sensory science to explore the molecular sensory basis of a specific flavor of CWT.

## Data availability statement

The data presented in the study are deposited in the OMIX, China National Center for Bioinformation/Beijing Institute of Genomics, Chinese Academy of Sciences, accession number OMIX002501, https://ngdc.cncb.ac.cn/omix/select-edit/OMIX002501.

## Author contributions

WS, ZhihuiW, ZhihuaW, FL, YH, and XL conceived and designed the experiments. ZhihuiW, HD, and ZhihuaW performed the experiments. ZhihuiW analyzed the data and wrote the manscript. HD, SW, and BS revised the manuscript critically. WS administrated the project. All the authors read and approved the final manuscript.
