# Supplementary material for: Identification of characteristic aroma and bacteria related to aroma evolution during long-term storage of compressed white tea
Source: Front Nutr. 2022 Dec 19;9:1092048. doi: 10.3389/fnut.2022.1092048 (PMC9806140; doi:10.3389/fnut.2022.1092048)
Supplement: Supplementary file 1 [file Data_Sheet_1.docx]

Table S1 Details of experimental samples

| Sample number | Production year / year | Storage time / year | Fresh leaf grade | Manufacturing season | Number of samples | Shape | Production area |
| --- | --- | --- | --- | --- | --- | --- | --- |
| A1 | 2018 | 1 | One bud, three leaves and four leaves | Spring | 3 | Round cake | Fuding City, Fujian Province, China |
| A3 | 2016 | 3 | One bud, three leaves and four leaves | Spring | 3 | Round cake | Fuding City, Fujian Province, China |
| A5 | 2014 | 5 | One bud, three leaves and four leaves | Spring | 3 | Round cake | Fuding City, Fujian Province, China |
| A7 | 2012 | 7 | One bud, three leaves and four leaves | Spring | 3 | Round cake | Fuding City, Fujian Province, China |
| A9 | 2010 | 9 | One bud, three leaves and four leaves | Spring | 3 | Round cake | Fuding City, Fujian Province, China |
| A10 | 2009 | 10 | One bud, three leaves and four leaves | Spring | 3 | Round cake | Fuding City, Fujian Province, China |
| A13 | 2006 | 13 | One bud, three leaves and four leaves | Spring | 3 | Round cake | Fuding City, Fujian Province, China |
| A16 | 2003 | 16 | One bud, three leaves and four leaves | Spring | 3 | Round cake | Fuding City, Fujian Province, China |

Table S2 Identification and relative content of volatile components

| Code | Classification | Volatile components | Odor description | CAS | Formula | RI | Dt | Identification | Relative content (%) | | | | | | | | Significance |
| --- | --- | --- | --- | --- | --- | --- | --- | --- | --- | --- | --- | --- | --- | --- | --- | --- | --- |
|  |  |  |  |  |  |  |  |  | A1 | A3 | A5 | A7 | A9 | A10 | A13 | A16 |  |
| 1 | Esters (5) | Methyl salicylate | Floral, fresh, minty, wintergreen-like | C119368 | C_8_H_8_O_3_ | 1164 | 1.1973 | RI, Dt | 2.22±0.1 | 0.51±0.03 | 0.53±0.03 | 0.61±0.04 | 0.43±0.02 | 0.46±0.03 | 0.48±0.01 | 0.66±0.03 | <0.001 |
| 2 |  | Ethyl benzoate | Green, fruity, balsamic, woody, fresh | C93890 | C_9_H_10_O_2_ | 1155 | 1.2594 | RI, Dt | 9.51±0.4 | 4.32±0.15 | 5.12±0.74 | 8.12±0.21 | 4.35±0.15 | 5.63±0.17 | 4.15±0.45 | 3.89±0.07 | <0.001 |
| 3 |  | γ-Butyrolactone D | Caramel, cream, milk, fatty, weak sweet | C96480 | C_4_H_6_O_2_ | 931 | 1.301 | RI, Dt | 13.79±0.04 | 14.85±0.16 | 17.55±2.26 | 16.37±0.3 | 28.26±0.11 | 29.44±0.95 | 25.02±1.08 | 16.69±2.23 | <0.001 |
| 4 |  | γ-Butyrolactone M | Caramel, cream, milk, fatty, weak sweet | C96480 | C_4_H_6_O_2_ | 930.9 | 1.0811 | RI, Dt | 3.67±0.08 | 3.7±0.06 | 3.62±0.29 | 2.8±0.09 | 2.27±0.03 | 2.27±0.02 | 2.42±0.03 | 3.7±0.19 | <0.001 |
| 5 |  | Butyl acetate | Fruity odor | C123864 | C_6_H_12_O_2_ | 818.2 | 1.242 | RI, Dt | 1.96±0.11 | 0.39±0.03 | 0.81±0.22 | 2.49±0.08 | 1.2±0.17 | 1.95±0.1 | 1.47±0.03 | 1.37±0.1 | <0.001 |
| 6 | Aldehydes (19) | (E)-2-Octenal | Herbal, fatty, green, citrus, cucumber-like | C2548870 | C_8_H_14_O | 1070.1 | 1.3271 | RI, Dt | 0.29±0.02 | 0.51±0.01 | 0.52±0.02 | 0.57±0.01 | 0.56±0.04 | 0.45±0.04 | 0.64±0.03 | 0.87±0.11 | <0.001 |
| 7 |  | Nonanal M | Citrus, rose, fatty, weak sweet | C124196 | C_9_H_18_O | 1103.4 | 1.4776 | RI, Dt | 0.37±0.01 | 0.5±0.03 | 0.55±0.04 | 0.41±0.01 | 0.43±0.02 | 0.38±0.01 | 0.47±0.05 | 0.73±0.05 | <0.001 |
| 8 |  | Nonanal D | Citrus, rose, fatty, weak sweet | C124196 | C_9_H_18_O | 1101.9 | 1.9464 | RI, Dt | 0.05±0 | 0.06±0 | 0.07±0.01 | 0.05±0 | 0.05±0 | 0.05±0.01 | 0.06±0 | 0.1±0.01 | <0.001 |
| 9 |  | Phenylacetaldehyde | Fruity, cherry-like, sweet | C122781 | C_8_H_8_O | 1056.8 | 1.2519 | RI, Dt | 3.7±0.52 | 3.97±0.25 | 3.79±0.2 | 3.22±0.12 | 2.13±0.11 | 2.22±0.17 | 2.86±0.2 | 2.68±0.11 | <0.001 |
| 10 |  | (E,E)-2,4-Heptadienal | Oily, coarseness smell | C4313035 | C_7_H_10_O | 1029.1 | 1.1962 | RI, Dt | 0.67±0.04 | 0.96±0.04 | 0.62±0.07 | 0.5±0.01 | 0.46±0.01 | 0.44±0.04 | 0.65±0.03 | 1.04±0.13 | <0.001 |
| 11 |  | Octanal D | Fatty, fruity, waxy | C124130 | C_8_H_16_O | 1021.2 | 1.8214 | RI, Dt | 0.08±0.01 | 0.11±0 | 0.09±0.01 | 0.1±0 | 0.08±0.01 | 0.07±0.01 | 0.11±0.02 | 0.17±0.03 | <0.001 |
| 12 |  | Octanal M | Herbal, almond-like, nutty | C124130 | C_8_H_16_O | 1021.4 | 1.421 | RI, Dt | 0.79±0.05 | 0.91±0.02 | 0.82±0.06 | 0.67±0.04 | 0.59±0.02 | 0.58±0.02 | 0.76±0.06 | 1.08±0.09 | <0.001 |
| 13 |  | Benzaldehyde M | Herbal, almond-like, nutty | C100527 | C_7_H_6_O | 974.5 | 1.1512 | RI, Dt | 1.84±0.05 | 2.17±0.03 | 2.3±0.2 | 2.07±0.05 | 1.88±0.03 | 1.66±0.01 | 1.83±0.03 | 2.34±0.07 | <0.001 |
| 14 |  | Benzaldehyde D | Sweet, fruity, cilantro | C100527 | C_7_H_6_O | 973.7 | 1.4697 | RI, Dt | 3.13±0.1 | 4.73±0.09 | 5.42±0.44 | 4.92±0.06 | 5.15±0.13 | 3.98±0.14 | 4.79±0.04 | 5.18±0.16 | <0.001 |
| 15 |  | (E)-2-Heptenal | Fruity, apple-like | C18829555 | C_7_H_12_O | 970.4 | 1.669 | RI, Dt | 0.19±0.01 | 0.31±0.02 | 0.36±0.04 | 0.78±0.13 | 0.16±0.02 | 0.16±0.03 | 0.82±0.3 | 1.18±0.25 | <0.001 |
| 16 |  | 3-Methylbutanal D | Fruity, bread-like | C590863 | C_5_H_10_O | 651.6 | 1.3997 | RI, Dt | 3.08±0.41 | 5.29±0.38 | 5.59±0.38 | 3.52±0.24 | 3.73±0.17 | 3.42±0.12 | 2.93±0.17 | 4.37±0.13 | <0.001 |
| 17 |  | 3-Methylbutanal M | Fruity, bread-like | C590863 | C_5_H_10_O | 646.7 | 1.1931 | RI, Dt | 0.81±0.15 | 0.81±0.02 | 0.91±0.14 | 1.27±0.19 | 0.45±0.05 | 0.59±0 | 1.28±0.29 | 1.41±0.18 | <0.001 |
| 18 |  | Pentanal D | Fatty, grass fragrance, apple-like | C110623 | C_5_H_10_O | 689.5 | 1.4226 | RI, Dt | 0.48±0.03 | 0.39±0.01 | 0.22±0.02 | 0.16±0.01 | 0.12±0.01 | 0.17±0.01 | 0.11±0.02 | 0.18±0.03 | <0.001 |
| 19 |  | Pentanal M | Fatty, grass fragrance, apple-like | C110623 | C_5_H_10_O | 688.3 | 1.1876 | RI, Dt | 0.46±0.08 | 0.5±0.03 | 0.37±0.05 | 0.21±0.02 | 0.17±0.02 | 0.2±0.02 | 0.18±0.02 | 0.3±0.02 | <0.001 |
| 20 |  | Hexanal M | Fatty, grass fragrance, apple-like | C66251 | C_6_H_12_O | 794.5 | 1.2585 | RI, Dt | 0.93±0.05 | 1±0.04 | 0.97±0.06 | 0.74±0.04 | 0.64±0.01 | 0.71±0.03 | 0.81±0.01 | 1.02±0.1 | <0.001 |
| 21 |  | Hexanal D | Green, oily, grassy, herbal, stale flavor | C66251 | C_6_H_12_O | 794 | 1.275 | RI, Dt | 0.54±0.01 | 0.62±0.06 | 0.63±0.12 | 0.26±0.02 | 0.3±0.02 | 0.36±0.01 | 0.57±0.05 | 0.46±0.1 | <0.001 |
| 22 |  | Hexanal T | Green, oily, grassy, herbal, stale flavor | C66251 | C_6_H_12_O | 794.2 | 1.5594 | RI, Dt | 1.48±0.09 | 1.98±0.12 | 1.73±0.09 | 1.81±0.05 | 1.82±0.04 | 2.02±0.04 | 2.43±0.2 | 3.59±0.36 | <0.001 |
| 23 |  | Heptanal M | Sweet, floral, fruity, woody | C111717 | C_7_H_14_O | 907.2 | 1.3413 | RI, Dt | 0.66±0.04 | 0.83±0.03 | 0.7±0.08 | 0.54±0.01 | 0.49±0.01 | 0.59±0.02 | 0.67±0.04 | 0.94±0.05 | <0.001 |
| 24 |  | Heptanal D | Sweet, floral, fruity, woody | C111717 | C_7_H_14_O | 906.4 | 1.6943 | RI, Dt | 0.16±0.02 | 0.19±0.03 | 0.17±0.03 | 0.15±0.01 | 0.16±0.01 | 0.18±0.02 | 0.21±0.03 | 0.39±0.09 | <0.001 |
| 25 | Alcohols (16) | Linalool M | Floral, woody, honey-like | C78706 | C_10_H_18_O | 1099.3 | 1.2168 | RI, Dt | 9.54±0.24 | 6.85±0.33 | 5.03±0.56 | 6.42±0.23 | 3.99±0.13 | 4.13±0.25 | 4.07±0.14 | 4.69±0.09 | <0.001 |
| 26 |  | Linalool D | Floral, woody, honey-like | C78706 | C_10_H_18_O | 1099.7 | 1.7557 | RI, Dt | 0.31±0.03 | 0.14±0.01 | 0.1±0.01 | 0.11±0.01 | 0.08±0 | 0.07±0.01 | 0.08±0.01 | 0.08±0.01 | <0.001 |
| 27 |  | Linalool oxide M | Mushroom, fresh, oily | C60047178 | C_10_H_18_O_2_ | 1078.5 | 1.26 | RI, Dt | 6.39±0.17 | 4.31±0.18 | 3.78±0.1 | 4.46±0.13 | 3.71±0.13 | 3.85±0.14 | 3.27±0.18 | 3.74±0.27 | <0.001 |
| 28 |  | Linalool oxide D | Apple-like, fruity | C60047178 | C_10_H_18_O_2_ | 1078.7 | 1.8115 | RI, Dt | 1.92±0.03 | 0.95±0.11 | 0.64±0.05 | 1.06±0.09 | 0.84±0.06 | 0.95±0.07 | 0.62±0.05 | 0.68±0.16 | <0.001 |
| 29 |  | 2-Phenylethanol D | Ethanol-like, pineapple, fruity | C60128 | C_8_H_10_O | 1111 | 1.5217 | RI, Dt | 0.89±0.03 | 0.31±0.02 | 0.3±0.03 | 0.36±0.03 | 0.19±0.01 | 0.14±0.01 | 0.19±0.01 | 0.18±0.01 | <0.001 |
| 30 |  | 2-Phenylethanol M | Ethanol-like, pineapple, fruity | C60128 | C_8_H_10_O | 1111.2 | 1.2949 | RI, Dt | 2.77±0.1 | 1.71±0.07 | 1.51±0.21 | 1.47±0.08 | 0.87±0.07 | 0.75±0.03 | 0.86±0.04 | 1.02±0.05 | <0.001 |
| 31 |  | 1-Octen-3-ol | Fruity, apple-like, spicy | C3391864 | C_8_H_16_O | 996.7 | 1.1576 | RI, Dt | 1.23±0.02 | 1.39±0.02 | 1.14±0.06 | 1.57±0.05 | 1.57±0.03 | 1.32±0.03 | 1.25±0.02 | 1.36±0.1 | <0.001 |
| 32 |  | Benzyl alcohol | Sweet, cherry-like, vanilla-like | C100516 | C_7_H_8_O | 1030.9 | 1.3244 | RI, Dt | 0.86±0.16 | 1.44±0.04 | 0.71±0.08 | 0.65±0.03 | 0.53±0.03 | 0.46±0.05 | 0.64±0.06 | 0.79±0.04 | <0.001 |
| 33 |  | (Z)-3-Hexen-1-ol | Herbal, banana-like, cheese | C928961 | C_6_H_12_O | 857.9 | 1.2398 | RI, Dt | 0.58±0.01 | 0.63±0.05 | 0.62±0.11 | 0.38±0.01 | 0.38±0.02 | 0.39±0.01 | 0.56±0.01 | 0.53±0.02 | <0.001 |
| 34 |  | 1-Propanol M | Herbal, banana-like, cheese | C71238 | C_3_H_8_O | 595.7 | 1.1108 | RI, Dt | 2.69±0.55 | 2.42±0.14 | 2.36±0.63 | 1.01±0.1 | 1.13±0.09 | 1.39±0.08 | 2.06±0.12 | 1.65±0.27 | <0.001 |
| 35 |  | 1-Propanol D | Fruity | C71238 | C_3_H_8_O | 597 | 1.2448 | RI, Dt | 3.32±0.76 | 7.52±0.74 | 8.44±0.61 | 8.39±0.76 | 8.56±0.43 | 8.44±0.18 | 7.99±0.21 | 8.55±0.39 | <0.001 |
| 36 |  | 2-Methyl-1-butanol D | Roasty, reminiscent of coffee and cocoa | C137326 | C_5_H_12_O | 763.9 | 1.4775 | RI, Dt | 0.45±0.1 | 0.54±0.01 | 0.44±0.02 | 0.67±0.01 | 0.77±0.02 | 0.6±0.04 | 0.56±0.01 | 0.67±0.08 | <0.001 |
| 37 |  | 2-Methyl-1-butanol M | Roasty, nutty, earthy | C137326 | C_5_H_12_O | 765.5 | 1.2332 | RI, Dt | 0.54±0.09 | 0.87±0.02 | 0.61±0.12 | 0.61±0.04 | 0.45±0.03 | 0.41±0.05 | 0.46±0.02 | 0.52±0.02 | <0.001 |
| 38 |  | 3-Methyl-1-butanol | Bean-like, fruity, earthy, fresh | C123513 | C_5_H_12_O | 734.7 | 1.1229 | RI, Dt | 0.81±0.14 | 0.92±0.03 | 0.98±0.19 | 0.75±0.05 | 0.75±0.07 | 0.77±0.04 | 0.96±0.08 | 0.5±0.07 | 0.003 |
| 39 |  | 1-Pentanol M | Citrus, lemon, orange-like, green | C71410 | C_5_H_12_O | 760.4 | 1.2491 | RI, Dt | 0.67±0.03 | 0.74±0.03 | 0.57±0.08 | 0.62±0.03 | 0.55±0 | 0.51±0.02 | 0.42±0.02 | 0.71±0.04 | <0.001 |
| 40 |  | 1-Pentanol D | Odor description | C71410 | C_5_H_12_O | 759.9 | 1.512 | RI, Dt | 0.49±0.04 | 0.71±0.03 | 0.45±0.05 | 0.67±0.02 | 1.12±0.03 | 0.71±0.03 | 0.45±0.01 | 0.64±0.09 | <0.001 |
| 41 | Ketones (7) | Acetophenone | Floral, fresh, minty, wintergreen-like | C98862 | C_8_H_8_O | 1068.9 | 1.1914 | RI, Dt | 1.38±0.03 | 1.13±0 | 1.31±0.08 | 1.57±0.05 | 1.55±0.04 | 1.4±0.04 | 1.3±0.04 | 1.37±0.04 | <0.001 |
| 42 |  | 2-Octanone | Green, fruity, balsamic, woody, fresh | C111137 | C_8_H_16_O | 1008.4 | 1.7512 | RI, Dt | 0.08±0 | 0.11±0 | 0.11±0.01 | 0.13±0 | 0.16±0.01 | 0.12±0.01 | 0.12±0.01 | 0.11±0.01 | <0.001 |
| 43 |  | 2-Heptanone M | Caramel, cream, milk, fatty, weak sweet | C110430 | C_7_H_14_O | 895.3 | 1.262 | RI, Dt | 0.7±0.03 | 0.89±0.01 | 0.8±0.04 | 0.77±0.01 | 0.71±0.01 | 0.71±0.02 | 0.68±0.01 | 0.96±0.04 | <0.001 |
| 44 |  | 2-Heptanone D | Caramel, cream, milk, fatty, weak sweet | C110430 | C_7_H_14_O | 893.5 | 1.6332 | RI, Dt | 0.88±0.12 | 1.63±0.1 | 1.41±0.07 | 2.2±0.19 | 2.53±0.15 | 2.36±0.16 | 1.86±0.09 | 1.69±0.31 | <0.001 |
| 45 |  | 3-Pentanone | Fruity odor | C96220 | C_5_H_10_O | 694.6 | 1.1035 | RI, Dt | 0.82±0.06 | 0.56±0.03 | 0.78±0.04 | 1.09±0.1 | 0.78±0.02 | 0.68±0.02 | 0.97±0.02 | 0.99±0.08 | <0.001 |
| 46 |  | Acetone | Herbal, fatty, green, citrus, cucumber-like | C67641 | C_3_H_6_O | 508 | 1.1134 | RI, Dt | 1.05±0.13 | 1.7±0.1 | 1.66±0.6 | 0.64±0.08 | 0.91±0.08 | 1.04±0.04 | 1.35±0.11 | 0.77±0.13 | 0.002 |
| 47 |  | 3-Octanone | Citrus, rose, fatty, weak sweet | C106683 | C_8_H_16_O | 967.9 | 1.3083 | RI, Dt | 0.27±0.02 | 0.32±0.03 | 0.36±0.06 | 0.22±0.01 | 0.27±0.01 | 0.24±0.03 | 0.37±0.01 | 0.3±0.03 | 0.001 |
| 48 | Heterocyclics (7) | 2-Ethyl-6-methylpyrazine | Citrus, rose, fatty, weak sweet | C13925036 | C_7_H_10_N_2_ | 1004.9 | 1.1743 | RI, Dt | 4.45±0.16 | 6.18±0.11 | 4.83±0.42 | 4.74±0.06 | 4.6±0.07 | 4.18±0.16 | 4.71±0.12 | 5.18±0.81 | 0.001 |
| 49 |  | Trimethylpyrazine | Fruity, cherry-like, sweet | C14667551 | C_7_H_10_N_2_ | 1017 | 1.6275 | RI, Dt | 0.05±0.01 | 0.09±0.01 | 0.06±0 | 0.08±0 | 0.05±0.01 | 0.04±0 | 0.08±0.01 | 0.09±0.02 | <0.001 |
| 50 |  | 2,6-Dimethylpyrazine | Oily, coarseness smell | C108509 | C_6_H_8_N_2_ | 918.8 | 1.1178 | RI, Dt | 0.49±0.43 | 0.21±0 | 0.23±0.02 | 0.27±0 | 0.32±0.01 | 0.24±0.01 | 0.31±0.02 | 0.24±0.01 | 0.675 |
| 51 |  | Methylpyrazine D | Fatty, fruity, waxy | C109080 | C_5_H_6_N_2_ | 829.5 | 1.393 | RI, Dt | 0.51±0.47 | 0.26±0.01 | 0.3±0.04 | 0.63±0.01 | 0.46±0.01 | 0.39±0.01 | 0.3±0.01 | 0.2±0.02 | 0.242 |
| 52 |  | Methylpyrazine M | Herbal, almond-like, nutty | C109080 | C_5_H_6_N_2_ | 831.3 | 1.0895 | RI, Dt | 3.32±0.73 | 3.99±0.08 | 5.09±0.44 | 4.2±0.15 | 4.67±0.12 | 4.3±0.13 | 5.92±0.23 | 5.22±0.31 | <0.001 |
| 53 |  | 2-Pentyl furan | Herbal, almond-like, nutty | C3777693 | C_9_H_14_O | 967.7 | 1.2556 | RI, Dt | 0.49±0.04 | 0.61±0.01 | 0.62±0.07 | 0.74±0.05 | 0.31±0.02 | 0.29±0.03 | 0.67±0.15 | 1.01±0.08 | <0.001 |
| 54 |  | 2-Acetylfuran | Sweet, fruity, cilantro | C1192627 | C_6_H_6_O_2_ | 905.9 | 1.4419 | RI, Dt | 0.08±0.01 | 0.14±0 | 0.21±0.04 | 0.25±0.02 | 0.34±0.01 | 0.25±0.02 | 0.35±0.03 | 0.44±0.03 | <0.001 |
| 55 | Terpenes (2) | Limonene | Fruity, apple-like | C138863 | C_10_H_16_ | 1057.3 | 1.2201 | RI, Dt | 1.03±0.03 | 0.91±0.02 | 0.89±0.06 | 0.74±0.04 | 0.78±0.03 | 0.74±0.03 | 0.67±0.02 | 0.59±0.01 | <0.001 |
| 56 |  | Styrene | Fruity, bread-like | C100425 | C_8_H_8_ | 893.3 | 1.4179 | RI, Dt | 0.13±0 | 0.19±0.01 | 0.19±0.01 | 0.19±0 | 0.19±0.01 | 0.16±0.01 | 0.18±0.01 | 0.17±0.01 | <0.001 |

Note: RI: retention index; Dt: represents the drift time in the drift tube; M: represents the monomer of the substance; D: represents the dimer of the substance; T: represents the trimer of the substance. The odor description referred to the literatures (22, 25, 36, 37).

Table S3 Correlation analysis of aroma sub-attributes and volatile components

| Aroma type | Volatile components | Correlation coefficient | Aroma type | Volatile components | Correlation coefficient | Aroma type | Volatile components | Correlation coefficient | Aroma type | Volatile components | Correlation coefficient | Aroma type | Volatile components | Correlation coefficient | Aroma type | Volatile components | Correlation coefficient |
| --- | --- | --- | --- | --- | --- | --- | --- | --- | --- | --- | --- | --- | --- | --- | --- | --- | --- |
| Sweet | Methyl salicylate | 0.658* | Floral | γ-Butyrolactone D | -0.626* | Fruity | (E)-2-Heptenal | -0.701* | Stale flavor | Pentanal M | -0.860** | Woody | Hexanal M | -0.695* | Herbal | (E)-2-Heptenal | 0.66* |
| Sweet | Ethyl benzoate | 0.672* | Floral | γ-Butyrolactone M | 0.62* | Fruity | Pentanal D | 0.744* | Stale flavor | Linalool M | -0.923** | Woody | Hexanal D | -0.68* | Herbal | Pentanal D | -0.712* |
| Sweet | γ-Butyrolactone D | -0.63* | Floral | (E)-2-Octenal | -0.663* | Fruity | Pentanal M | 0.683* | Stale flavor | Linalool D | -0.941** | Woody | Linalool M | -0.856** | Herbal | Pentanal M | -0.651* |
| Sweet | (E)-2-Octenal | -0.764* | Floral | Phenylacetaldehyde | 0.878** | Fruity | Hexanal T | -0.759* | Stale flavor | Linalool oxide M | -0.865** | Woody | Linalool D | -0.833* | Herbal | Hexanal T | 0.857** |
| Sweet | Phenylacetaldehyde | 0.77* | Floral | Pentanal D | 0.785* | Fruity | Heptanal D | -0.677* | Stale flavor | Linalool oxide D | -0.774* | Woody | Linalool oxide M | -0.744* | Herbal | Heptanal D | 0.746* |
| Sweet | Pentanal D | 0.862** | Floral | Pentanal M | 0.842** | Fruity | Linalool M | 0.662* | Stale flavor | 1-Propanol M | -0.808* | Woody | Linalool oxide D | -0.633* | Herbal | Linalool M | -0.729* |
| Sweet | Pentanal M | 0.748* | Floral | Hexanal T | -0.645* | Fruity | Benzyl alcohol | 0.631* | Stale flavor | 1-Propanol D | 0.904** | Woody | Benzyl alcohol | -0.757* | Herbal | Linalool D | -0.654* |
| Sweet | Hexanal T | -0.701* | Floral | Linalool M | 0.697* | Fruity | Methylpyrazine M | -0.714* | Stale flavor | 2-Heptanone D | 0.889** | Woody | 1-Propanol M | -0.868** | Herbal | Linalool oxide M | -0.655* |
| Sweet | Linalool M | 0.91** | Floral | Benzyl alcohol | 0.634* | Fruity | Limonene | 0.879** | Stale flavor | Methylpyrazine M | 0.624* | Woody | 1-Propanol D | 0.785* | Herbal | Methylpyrazine M | 0.709* |
| Sweet | Linalool D | 0.841** | Floral | 1-Propanol M | 0.672* | Stale flavor | Methyl salicylate | -0.853** | Stale flavor | Limonene | -0.812** | Woody | 2-Heptanone D | 0.877** | Herbal | Limonene | -0.936** |
| Sweet | Linalool oxide M | 0.831* | Floral | 2-Heptanone D | -0.621* | Stale flavor | γ-Butyrolactone D | 0.731* | Woody | Methyl salicylate | -0.699* | Woody | Limonene | -0.801* | Sweet | 2-Phenylethanol M | 0.909** |
| Sweet | Linalool oxide D | 0.768* | Floral | Limonene | 0.902** | Stale flavor | γ-Butyrolactone M | -0.716* | Woody | γ-Butyrolactone D | 0.754* | Woody | Pentanal M | -0.954** | Sweet | 2-Acetylfuran | -0.931** |
| Sweet | 1-Propanol D | -0.75* | Fruity | (E)-2-Octenal | -0.791* | Stale flavor | Phenylacetaldehyde | -0.761* | Woody | γ-Butyrolactone M | -0.787* | Herbal | (E)-2-Octenal | 0.835* | Sweet | 2-Phenylethanol D | 0.835* |
| Sweet | Methylpyrazine M | -0.797* | Fruity | Nonanal D | -0.616* | Stale flavor | Benzaldehyde D | 0.601* | Woody | Phenylacetaldehyde | -0.842** | Herbal | Nonanal D | 0.617* | Floral | 2-Phenylethanol M | 0.769* |
| Sweet | Limonene | 0.911** | Fruity | Phenylacetaldehyde | 0.665* | Stale flavor | Pentanal D | -0.953** | Woody | Pentanal D | -0.970** | Herbal | Phenylacetaldehyde | -0.741* | Floral | 2-Acetylfuran | -0.891** |
| Floral | 2-Methyl-1-butanol D | -0.715* | Stale flavor | 2-Phenylethanol M | -0.962** | Stale flavor | 2-Phenylethanol D | -0.910** | Woody | 2-Phenylethanol M | -0.896** | Woody | 2-Phenylethanol D | -0.782* | Woody | 2-Octanone | 0.776* |
| Fruity | 2-Acetylfuran | -0.921** | Stale flavor | 2-Acetylfuran | 0.762* | Stale flavor | 2-Octanone | 0.762* | Woody | 2-Acetylfuran | 0.777* | Woody | 2-Methyl-1-butanol D | 0.742* | Woody | (Z)-3-Hexen-1-ol | -0.765* |
| Woody | 1-Pentanol M | -0.628* | Herbal | 2-Phenylethanol M | -0.767* | Herbal | 2-Acetylfuran | 0.951** |  |  |  |  |  |  |  |  |  |

Note: ** *P* < 0.01; * *P* < 0.05.

Table S4 Bacterial community of compressed white tea during storage

| Genus | A1 | A5 | A9 | A10 | A13 | A16 |
| --- | --- | --- | --- | --- | --- | --- |
| g__Oxyphotobacteria_unclassified | 80.767 | 81.741 | 93.144 | 59.623 | 43.209 | 9.025 |
| g__Sphingomonas | 1.922 | 2.805 | 0.382 | 19.845 | 0.467 | 8.735 |
| g__Brevundimonas | 0.002 | 0.023 | 0.086 | 0.026 | 0.003 | 5.899 |
| g__Lawsonella | 0.000 | 0.000 | 0.066 | 0.031 | 0.057 | 3.271 |
| g__Corynebacterium_1 | 0.000 | 0.002 | 0.006 | 0.014 | 0.116 | 3.244 |
| g__Schlegelella | 0.000 | 0.000 | 0.051 | 0.000 | 0.000 | 3.205 |
| g__Streptococcus | 0.148 | 0.038 | 0.012 | 0.077 | 0.020 | 2.652 |
| g__Staphylococcus | 0.009 | 0.051 | 0.010 | 0.118 | 0.181 | 2.616 |
| g__Corynebacterium | 0.036 | 0.002 | 0.090 | 0.000 | 0.234 | 2.605 |
| g__Paenibacillus | 0.000 | 0.020 | 0.000 | 0.010 | 0.058 | 2.351 |
| g__Burkholderia | 0.286 | 0.033 | 0.057 | 0.005 | 0.198 | 2.169 |
| g__Bacillus | 0.855 | 0.022 | 0.002 | 0.128 | 0.061 | 2.093 |
| g__Ralstonia | 0.157 | 0.000 | 0.000 | 0.070 | 0.003 | 2.072 |
| g__Lactobacillus | 0.000 | 0.050 | 0.072 | 0.102 | 0.030 | 2.032 |
| g__Fusobacterium | 0.000 | 0.000 | 0.013 | 0.000 | 0.000 | 1.964 |
| g__Acinetobacter | 0.008 | 0.172 | 0.045 | 0.100 | 0.080 | 1.949 |
| g__Enterococcus | 0.000 | 0.000 | 0.000 | 0.000 | 0.001 | 1.856 |
| g__Bacteroides | 0.000 | 0.004 | 0.000 | 0.079 | 0.072 | 1.790 |
| g__Methylobacterium | 0.185 | 2.721 | 0.132 | 2.024 | 0.308 | 1.594 |
| g__Novosphingobium | 0.000 | 0.004 | 0.000 | 0.000 | 0.012 | 1.447 |
| g__Actinobacteria_unclassified | 0.000 | 0.000 | 0.008 | 0.000 | 0.226 | 1.445 |
| g__Klebsiella | 0.000 | 0.007 | 0.000 | 0.100 | 0.069 | 1.329 |
| g__Methyloversatilis | 0.000 | 0.000 | 0.000 | 0.000 | 0.000 | 1.323 |
| g__Escherichia-Shigella | 0.001 | 0.017 | 0.037 | 0.114 | 0.178 | 1.172 |
| g__Enhydrobacter | 0.037 | 0.022 | 0.015 | 0.007 | 0.000 | 1.031 |
| g__Cutibacterium | 0.000 | 0.003 | 0.000 | 0.015 | 0.000 | 0.976 |
| g__Actinomyces | 0.000 | 0.000 | 0.000 | 0.000 | 0.020 | 0.946 |
| g__Pseudomonas | 0.006 | 0.020 | 0.000 | 0.053 | 0.033 | 0.885 |
| g__Mitochondria_unclassified | 10.686 | 6.870 | 5.239 | 2.751 | 2.570 | 0.719 |
| g__Odoribacter | 0.000 | 0.000 | 0.000 | 0.000 | 0.000 | 0.707 |
| g__Abiotrophia | 0.000 | 0.000 | 0.000 | 0.000 | 0.000 | 0.689 |
| g__Cloacibacterium | 0.000 | 0.000 | 0.000 | 0.000 | 0.001 | 0.668 |
| g__Subgroup_7_unclassified | 0.000 | 0.000 | 0.000 | 0.000 | 0.000 | 0.644 |
| g__Micrococcus | 0.000 | 0.000 | 0.025 | 0.000 | 0.000 | 0.615 |
| g__Phyllobacterium | 0.000 | 0.000 | 0.000 | 0.000 | 0.000 | 0.552 |
| g__Anaerococcus | 0.000 | 0.000 | 0.000 | 0.054 | 0.000 | 0.528 |
| g__Lachnoanaerobaculum | 0.000 | 0.000 | 0.000 | 0.000 | 0.000 | 0.497 |
| g__Vulcaniibacterium | 0.000 | 0.000 | 0.000 | 0.000 | 0.000 | 0.488 |
| g__Paracoccus | 0.000 | 0.032 | 0.045 | 0.000 | 0.117 | 0.482 |
| g__Leptotrichia | 0.000 | 0.000 | 0.000 | 0.000 | 0.000 | 0.422 |
| g__Actinomycetales_unclassified | 0.000 | 0.000 | 0.000 | 0.000 | 0.000 | 0.421 |
| g__Acidovorax | 0.066 | 0.634 | 0.000 | 0.728 | 0.030 | 0.412 |
| g__Helicobacter | 0.000 | 0.000 | 0.000 | 0.000 | 0.008 | 0.405 |
| g__Stenotrophomonas | 0.000 | 0.018 | 0.003 | 0.000 | 0.115 | 0.391 |
| g__Devosia | 0.000 | 0.021 | 0.000 | 0.035 | 0.000 | 0.388 |
| g__Peptoniphilus | 0.000 | 0.000 | 0.000 | 0.000 | 0.000 | 0.365 |
| g__Lachnoclostridium | 0.000 | 0.000 | 0.000 | 0.000 | 0.000 | 0.355 |
| g__Shinella | 0.000 | 0.000 | 0.000 | 0.000 | 0.002 | 0.344 |
| g__Chryseobacterium | 0.013 | 0.224 | 0.000 | 0.063 | 0.090 | 0.343 |
| g__Parabacteroides | 0.001 | 0.000 | 0.000 | 0.000 | 0.003 | 0.338 |
| g__Proteiniphilum | 0.049 | 0.000 | 0.000 | 0.000 | 0.000 | 0.332 |
| g__Eubacterium]_coprostanoligenes_group | 0.000 | 0.000 | 0.000 | 0.000 | 0.038 | 0.321 |
| g__Selenomonas | 0.000 | 0.000 | 0.000 | 0.000 | 0.000 | 0.318 |
| g__Bradyrhizobium | 0.000 | 0.027 | 0.002 | 0.012 | 0.003 | 0.316 |
| g__Lautropia | 0.000 | 0.000 | 0.027 | 0.000 | 0.031 | 0.313 |
| g__Intestinibacter | 0.000 | 0.000 | 0.000 | 0.000 | 0.000 | 0.300 |
| g__Megasphaera | 0.000 | 0.000 | 0.000 | 0.000 | 0.000 | 0.290 |
| g__Simplicispira | 0.000 | 0.000 | 0.000 | 0.000 | 0.000 | 0.289 |
| g__Atopobium | 0.000 | 0.000 | 0.000 | 0.000 | 0.000 | 0.286 |
| g__Beijerinckiaceae_unclassified | 0.000 | 0.038 | 0.000 | 0.000 | 0.000 | 0.279 |
| g__Clostridium_sensu_stricto_5 | 0.000 | 0.000 | 0.000 | 0.000 | 0.000 | 0.273 |
| g__Pseudoxanthomonas | 0.000 | 0.000 | 0.000 | 0.000 | 0.006 | 0.268 |
| g__Haemophilus | 0.013 | 0.000 | 0.006 | 0.000 | 0.000 | 0.245 |
| g__Erysipelothrix | 0.000 | 0.000 | 0.000 | 0.000 | 0.000 | 0.245 |
| g__Akkermansia | 0.000 | 0.000 | 0.000 | 0.052 | 0.212 | 0.245 |
| g__Bifidobacterium | 0.520 | 0.000 | 0.001 | 0.003 | 0.012 | 0.244 |
| g__Arthrobacter | 0.000 | 0.000 | 0.010 | 0.000 | 0.013 | 0.241 |
| g__Candidatus_Bacilloplasma | 0.025 | 0.000 | 0.000 | 0.000 | 0.000 | 0.239 |
| g__Pedobacter | 0.372 | 0.503 | 0.005 | 0.495 | 0.007 | 0.238 |
| g__Pelomonas | 0.000 | 0.000 | 0.000 | 0.024 | 0.007 | 0.227 |
| g__Sandaracinobacter | 0.000 | 0.000 | 0.000 | 0.000 | 0.000 | 0.220 |
| g__Gardnerella | 0.000 | 0.000 | 0.013 | 0.000 | 0.000 | 0.215 |
| g__Clavibacter | 0.000 | 0.000 | 0.030 | 0.000 | 0.000 | 0.215 |
| g__Candidatus_Hepatoplasma | 0.000 | 0.000 | 0.000 | 0.000 | 0.000 | 0.211 |
| g__Kocuria | 0.000 | 0.009 | 0.018 | 0.000 | 0.035 | 0.208 |
| g__Zymophilus | 0.000 | 0.000 | 0.000 | 0.000 | 0.000 | 0.207 |
| g__unclassified | 0.000 | 0.000 | 0.000 | 0.006 | 0.003 | 0.203 |
| g__Renibacterium | 0.000 | 0.000 | 0.000 | 0.000 | 0.019 | 0.203 |
| g__Hungatella | 0.000 | 0.000 | 0.000 | 0.000 | 0.000 | 0.199 |
| g__Hydrogenophaga | 0.000 | 0.000 | 0.000 | 0.000 | 0.006 | 0.184 |
| g__Neisseria | 0.037 | 0.000 | 0.000 | 0.007 | 0.000 | 0.184 |
| g__Ruminococcus]_torques_group | 0.000 | 0.004 | 0.000 | 0.000 | 0.074 | 0.184 |
| g__Prevotella | 0.024 | 0.000 | 0.000 | 0.000 | 0.000 | 0.182 |
| g__Ruminococcaceae_NK4A214_group | 0.000 | 0.000 | 0.000 | 0.000 | 0.027 | 0.179 |
| g__Campylobacter | 0.000 | 0.000 | 0.000 | 0.000 | 0.104 | 0.177 |
| g__Sphingopyxis | 0.000 | 0.000 | 0.000 | 0.000 | 0.007 | 0.176 |
| g__Brachybacterium | 0.000 | 0.023 | 0.000 | 0.000 | 0.000 | 0.175 |
| g__Cellulomonas | 0.000 | 0.003 | 0.000 | 0.000 | 0.000 | 0.174 |
| g__Aeromonas | 0.000 | 0.013 | 0.000 | 0.004 | 0.011 | 0.169 |
| g__Amaricoccus | 0.000 | 0.000 | 0.000 | 0.000 | 0.000 | 0.169 |
| g__Luteimonas | 0.004 | 0.000 | 0.000 | 0.000 | 0.000 | 0.166 |
| g__Ruminococcaceae_UCG-002 | 0.000 | 0.000 | 0.000 | 0.000 | 0.000 | 0.166 |
| g__Veillonellaceae_unclassified | 0.000 | 0.000 | 0.000 | 0.000 | 0.001 | 0.161 |
| g__Lutispora | 0.000 | 0.000 | 0.000 | 0.000 | 0.000 | 0.160 |
| g__Subgroup_6_unclassified | 0.000 | 0.002 | 0.000 | 0.062 | 0.003 | 0.159 |
| g__Rothia | 0.000 | 0.000 | 0.000 | 0.000 | 0.039 | 0.158 |
| g__Enterobacter | 0.000 | 0.043 | 0.009 | 0.067 | 0.000 | 0.146 |
| g__Weeksellaceae_unclassified | 0.000 | 0.000 | 0.010 | 0.000 | 0.000 | 0.145 |
| g__Ramlibacter | 0.000 | 0.000 | 0.000 | 0.000 | 0.000 | 0.145 |
| g__Clostridium_sensu_stricto_12 | 0.000 | 0.000 | 0.000 | 0.000 | 0.000 | 0.145 |
| g__Microbacteriaceae_unclassified | 1.384 | 0.026 | 0.000 | 0.000 | 0.000 | 0.143 |
| g__Comamonas | 0.000 | 0.020 | 0.002 | 0.000 | 0.000 | 0.143 |
| g__Citrobacter | 0.000 | 0.000 | 0.000 | 0.000 | 0.000 | 0.140 |
| g__Aliterella_CENA595 | 0.000 | 0.006 | 0.000 | 0.028 | 0.000 | 0.137 |
| g__Delftia | 0.000 | 0.000 | 0.028 | 0.000 | 0.055 | 0.133 |
| g__Peptococcaceae_unclassified | 0.000 | 0.000 | 0.000 | 0.000 | 0.174 | 0.133 |
| g__Nitrosospira | 0.000 | 0.000 | 0.000 | 0.000 | 0.000 | 0.128 |
| g__Aureimonas | 0.908 | 0.563 | 0.010 | 3.498 | 0.090 | 0.128 |
| g__0319-6G20_unclassified | 0.170 | 0.000 | 0.000 | 0.000 | 0.000 | 0.122 |
| g__Sphingobacterium | 0.000 | 0.005 | 0.000 | 0.000 | 0.012 | 0.122 |
| g__Fusobacteriaceae_unclassified | 0.000 | 0.000 | 0.000 | 0.000 | 0.000 | 0.122 |
| g__Ruminococcaceae_UCG-014 | 0.000 | 0.000 | 0.000 | 0.000 | 0.015 | 0.118 |
| g__Hydrogenophilus | 0.000 | 0.000 | 0.000 | 0.000 | 0.000 | 0.118 |
| g__Parasegetibacter | 0.000 | 0.000 | 0.000 | 0.000 | 0.000 | 0.117 |
| g__hgcI_clade | 0.000 | 0.000 | 0.000 | 0.000 | 0.000 | 0.117 |
| g__Porphyrobacter | 0.000 | 0.000 | 0.000 | 0.004 | 0.000 | 0.113 |
| g__Merdimonas | 0.000 | 0.000 | 0.000 | 0.000 | 0.000 | 0.113 |
| g__Fretibacterium | 0.018 | 0.000 | 0.000 | 0.000 | 0.000 | 0.111 |
| g__Ignavigranum | 0.000 | 0.000 | 0.000 | 0.000 | 0.000 | 0.107 |
| g__Variovorax | 0.000 | 0.003 | 0.001 | 0.038 | 0.019 | 0.105 |
| g__Tessaracoccus | 0.000 | 0.000 | 0.000 | 0.000 | 0.000 | 0.104 |
| g__Ruminococcaceae_unclassified | 0.000 | 0.000 | 0.000 | 0.000 | 0.000 | 0.103 |
| g__Subdoligranulum | 0.000 | 0.000 | 0.000 | 0.000 | 0.000 | 0.099 |
| g__Rhodococcus | 0.000 | 0.000 | 0.000 | 0.000 | 0.000 | 0.099 |
| g__Aggregatibacter | 0.000 | 0.000 | 0.000 | 0.000 | 0.000 | 0.096 |
| g__Halomonas | 0.000 | 0.000 | 0.000 | 0.000 | 0.000 | 0.094 |
| g__Ottowia | 0.000 | 0.000 | 0.000 | 0.000 | 0.000 | 0.088 |
| g__Verticia | 0.000 | 0.000 | 0.000 | 0.000 | 0.000 | 0.088 |
| g__Anaerostipes | 0.000 | 0.000 | 0.000 | 0.000 | 0.000 | 0.086 |
| g__Saccharimonadales_unclassified | 0.000 | 0.000 | 0.000 | 0.000 | 0.000 | 0.086 |
| g__Qipengyuania | 0.000 | 0.000 | 0.000 | 0.000 | 0.000 | 0.085 |
| g__Oceanobacillus | 0.000 | 0.000 | 0.000 | 0.005 | 0.000 | 0.083 |
| g__Bergeyella | 0.000 | 0.000 | 0.001 | 0.000 | 0.000 | 0.081 |
| g__Prevotella_2 | 0.000 | 0.000 | 0.000 | 0.000 | 0.000 | 0.081 |
| g__Finegoldia | 0.000 | 0.000 | 0.000 | 0.000 | 0.000 | 0.080 |
| g__Aquabacterium | 0.000 | 0.000 | 0.002 | 0.037 | 0.027 | 0.079 |
| g__Ruminococcus]_gnavus_group | 0.000 | 0.000 | 0.000 | 0.000 | 0.006 | 0.078 |
| g__WD2101_soil_group_unclassified | 0.000 | 0.000 | 0.000 | 0.000 | 0.000 | 0.078 |
| g__Thermomonas | 0.000 | 0.000 | 0.024 | 0.000 | 0.037 | 0.076 |
| g__Faecalicoccus | 0.000 | 0.000 | 0.000 | 0.000 | 0.000 | 0.076 |
| g__Curvibacter | 0.000 | 0.020 | 0.006 | 0.000 | 0.000 | 0.075 |
| g__Eubacterium]_fissicatena_group | 0.000 | 0.000 | 0.000 | 0.000 | 0.000 | 0.075 |
| g__Empedobacter | 0.000 | 0.000 | 0.000 | 0.000 | 0.000 | 0.075 |
| g__Rubellimicrobium | 0.054 | 0.330 | 0.000 | 0.000 | 0.000 | 0.073 |
| g__Massilia | 0.000 | 0.003 | 0.009 | 0.018 | 0.000 | 0.072 |
| g__Roseomonas | 0.016 | 0.006 | 0.017 | 0.000 | 0.000 | 0.072 |
| g__Blautia | 0.000 | 0.003 | 0.000 | 0.000 | 0.000 | 0.072 |
| g__Anoxybacillus | 0.000 | 0.000 | 0.000 | 0.000 | 0.000 | 0.072 |
| g__Sphingobacteriaceae_unclassified | 0.000 | 0.000 | 0.000 | 0.000 | 0.000 | 0.070 |
| g__Lachnospiraceae_unclassified | 0.001 | 0.000 | 0.000 | 0.000 | 0.034 | 0.068 |
| g__Veillonella | 0.000 | 0.000 | 0.050 | 0.000 | 0.000 | 0.068 |
| g__Brevibacterium | 0.000 | 0.000 | 0.000 | 0.000 | 0.144 | 0.067 |
| g__Alistipes | 0.001 | 0.000 | 0.000 | 0.000 | 0.118 | 0.067 |
| g__67-14_unclassified | 0.000 | 0.000 | 0.000 | 0.000 | 0.000 | 0.067 |
| g__Aerococcus | 0.000 | 0.000 | 0.000 | 0.000 | 0.000 | 0.067 |
| g__Dadabacteriales_unclassified | 0.000 | 0.000 | 0.000 | 0.000 | 0.000 | 0.063 |
| g__Dialister | 0.000 | 0.000 | 0.001 | 0.000 | 0.000 | 0.062 |
| g__Prevotellaceae_unclassified | 0.000 | 0.000 | 0.000 | 0.000 | 0.000 | 0.059 |
| g__Bdellovibrio | 0.000 | 0.040 | 0.000 | 0.000 | 0.003 | 0.059 |
| g__Geodermatophilus | 0.000 | 0.005 | 0.000 | 0.000 | 0.000 | 0.056 |
| g__Kytococcus | 0.000 | 0.000 | 0.000 | 0.071 | 0.000 | 0.054 |
| g__Propionispira | 0.000 | 0.000 | 0.000 | 0.000 | 0.000 | 0.054 |
| g__Clostridium_sensu_stricto_1 | 0.000 | 0.001 | 0.000 | 0.000 | 0.049 | 0.052 |
| g__Clostridium]_innocuum_group | 0.000 | 0.000 | 0.000 | 0.000 | 0.000 | 0.052 |
| g__Actinomycetaceae_unclassified | 0.000 | 0.000 | 0.000 | 0.000 | 0.000 | 0.052 |
| g__Gemmatirosa | 0.000 | 0.007 | 0.000 | 0.000 | 0.000 | 0.049 |
| g__Lactococcus | 0.000 | 0.000 | 0.000 | 0.000 | 0.000 | 0.049 |
| g__Janibacter | 0.000 | 0.000 | 0.000 | 0.000 | 0.011 | 0.046 |
| g__Acidibacter | 0.000 | 0.000 | 0.000 | 0.000 | 0.001 | 0.046 |
| g__Nocardioides | 0.000 | 0.054 | 0.000 | 0.000 | 0.000 | 0.044 |
| g__Chroococcidiopsis_SAG_2023 | 0.000 | 0.000 | 0.000 | 0.000 | 0.000 | 0.044 |
| g__Xanthomonas | 0.000 | 0.000 | 0.000 | 0.000 | 0.000 | 0.041 |
| g__Clostridiales_vadinBB60_group_unclassified | 0.000 | 0.000 | 0.000 | 0.000 | 0.000 | 0.036 |
| g__Rhizobium | 0.073 | 0.006 | 0.000 | 0.149 | 0.000 | 0.033 |
| g__Caulobacteraceae_unclassified | 0.000 | 0.068 | 0.001 | 0.000 | 0.074 | 0.031 |
| g__Algoriphagus | 0.000 | 0.000 | 0.000 | 0.000 | 0.000 | 0.029 |
| g__Cardiobacterium | 0.000 | 0.000 | 0.000 | 0.000 | 0.000 | 0.026 |
| g__Nesterenkonia | 0.000 | 0.000 | 0.000 | 0.000 | 0.000 | 0.026 |
| g__Trichococcus | 0.000 | 0.000 | 0.000 | 0.000 | 0.000 | 0.026 |
| g__Cellvibrio | 0.000 | 0.000 | 0.000 | 0.000 | 0.000 | 0.024 |
| g__Tsukamurella | 0.000 | 0.000 | 0.000 | 0.000 | 0.000 | 0.023 |
| g__Gammaproteobacteria_unclassified | 0.000 | 0.011 | 0.000 | 0.000 | 0.000 | 0.021 |
| g__Microbacterium | 0.041 | 0.070 | 0.000 | 0.115 | 0.004 | 0.020 |
| g__Muribaculaceae_unclassified | 0.000 | 0.002 | 0.000 | 0.000 | 0.340 | 0.018 |
| g__A4b_unclassified | 0.000 | 0.000 | 0.000 | 0.000 | 0.000 | 0.018 |
| g__Albidovulum | 0.000 | 0.000 | 0.000 | 0.000 | 0.000 | 0.016 |
| g__Salinicoccus | 0.000 | 0.000 | 0.000 | 0.000 | 0.000 | 0.015 |
| g__Herbaspirillum | 0.143 | 0.018 | 0.000 | 0.088 | 0.000 | 0.014 |
| g__Sphingomonadaceae_unclassified | 0.000 | 0.006 | 0.000 | 0.036 | 0.000 | 0.014 |
| g__Kineococcus | 0.000 | 0.008 | 0.000 | 0.000 | 0.000 | 0.013 |
| g__Weissella | 0.234 | 0.001 | 0.000 | 0.000 | 0.000 | 0.011 |
| g__Bosea | 0.122 | 0.158 | 0.006 | 0.159 | 0.061 | 0.010 |
| g__Capnocytophaga | 0.000 | 0.000 | 0.000 | 0.000 | 0.000 | 0.010 |
| g__Actinomycetospora | 0.000 | 0.004 | 0.000 | 0.000 | 1.674 | 0.010 |
| g__Taibaiella | 0.000 | 0.000 | 0.000 | 0.000 | 0.000 | 0.007 |
| g__Flavisolibacter | 0.000 | 0.000 | 0.000 | 0.000 | 0.000 | 0.006 |
| g__Pseudoramibacter | 0.000 | 0.000 | 0.000 | 0.000 | 0.002 | 0.005 |
| g__Blastococcus | 0.000 | 0.000 | 0.000 | 0.000 | 0.000 | 0.005 |
| g__Gemmatimonas | 0.000 | 0.000 | 0.001 | 0.000 | 0.000 | 0.005 |
| g__Eubacterium]_oxidoreducens_group | 0.000 | 0.000 | 0.000 | 0.000 | 0.000 | 0.005 |
| g__Succiniclasticum | 0.000 | 0.000 | 0.000 | 0.000 | 0.049 | 0.004 |
| g__Dermabacter | 0.000 | 0.000 | 0.000 | 0.000 | 0.000 | 0.004 |
| g__SWB02 | 0.000 | 0.000 | 0.000 | 0.000 | 0.000 | 0.003 |
| g__Allorhizobium | 0.073 | 0.148 | 0.000 | 7.233 | 0.229 | 0.003 |
| g__Exiguobacterium | 0.000 | 0.001 | 0.000 | 0.000 | 0.000 | 0.003 |
| g__Saccharofermentans | 0.000 | 0.000 | 0.000 | 0.000 | 0.000 | 0.003 |
| g__Noviherbaspirillum | 0.000 | 0.000 | 0.002 | 0.000 | 0.000 | 0.003 |
| g__Kroppenstedtia | 0.000 | 0.000 | 0.000 | 0.000 | 0.000 | 0.002 |
| g__Streptomyces | 0.000 | 0.028 | 0.001 | 0.000 | 11.322 | 0.000 |
| g__Saccharomonospora | 0.000 | 0.000 | 0.000 | 0.000 | 7.800 | 0.000 |
| g__Stackebrandtia | 0.000 | 0.000 | 0.000 | 0.000 | 6.618 | 0.000 |
| g__Saccharopolyspora | 0.000 | 0.003 | 0.004 | 0.000 | 5.248 | 0.000 |
| g__Prauserella | 0.000 | 0.000 | 0.000 | 0.000 | 4.547 | 0.000 |
| g__Allostreptomyces | 0.000 | 0.000 | 0.000 | 0.000 | 3.168 | 0.000 |
| g__Pseudonocardia | 0.000 | 0.002 | 0.000 | 0.000 | 3.034 | 0.000 |
| g__Nocardia | 0.000 | 0.000 | 0.000 | 0.000 | 2.123 | 0.000 |
| g__Actinokineospora | 0.000 | 0.000 | 0.000 | 0.000 | 0.761 | 0.000 |
| g__Kineosporia | 0.000 | 0.000 | 0.000 | 0.000 | 0.521 | 0.000 |
| g__Actinomadura | 0.000 | 0.000 | 0.000 | 0.000 | 0.369 | 0.000 |
| g__Amycolatopsis | 0.000 | 0.000 | 0.000 | 0.000 | 0.230 | 0.000 |
| g__Actinocatenispora | 0.000 | 0.000 | 0.000 | 0.000 | 0.219 | 0.000 |
| g__Comamonadaceae_unclassified | 0.000 | 0.000 | 0.000 | 0.000 | 0.175 | 0.000 |
| g__Betaproteobacteria_unclassified | 0.000 | 0.000 | 0.000 | 0.000 | 0.130 | 0.000 |
| g__Eisenbergiella | 0.000 | 0.000 | 0.000 | 0.000 | 0.095 | 0.000 |
| g__Ruminiclostridium_9 | 0.000 | 0.000 | 0.000 | 0.000 | 0.086 | 0.000 |
| g__Hyphomonadaceae_unclassified | 0.000 | 0.000 | 0.000 | 0.000 | 0.080 | 0.000 |
| g__Olsenella | 0.000 | 0.000 | 0.000 | 0.000 | 0.074 | 0.000 |
| g__Sciscionella | 0.000 | 0.000 | 0.000 | 0.000 | 0.065 | 0.000 |
| g__Thalassospira | 0.000 | 0.000 | 0.000 | 0.000 | 0.062 | 0.000 |
| g__Mycobacterium | 0.000 | 0.000 | 0.000 | 0.000 | 0.058 | 0.000 |
| g__Tolumonas | 0.000 | 0.000 | 0.000 | 0.000 | 0.049 | 0.000 |
| g__Lachnospiraceae_NK3A20_group | 0.000 | 0.000 | 0.000 | 0.000 | 0.040 | 0.000 |
| g__Cohnella | 0.000 | 0.000 | 0.000 | 0.000 | 0.036 | 0.000 |
| g__Aquicella | 0.000 | 0.000 | 0.000 | 0.000 | 0.036 | 0.000 |
| g__Prevotella_6 | 0.000 | 0.000 | 0.000 | 0.000 | 0.032 | 0.000 |
| g__Dechloromonas | 0.000 | 0.000 | 0.008 | 0.000 | 0.032 | 0.000 |
| g__Alicycliphilus | 0.000 | 0.000 | 0.000 | 0.000 | 0.031 | 0.000 |
| g__Gemmobacter | 0.000 | 0.000 | 0.000 | 0.000 | 0.030 | 0.000 |
| g__Mucilaginibacter | 0.000 | 0.029 | 0.000 | 0.000 | 0.026 | 0.000 |
| g__Succinivibrionaceae_UCG-001 | 0.000 | 0.000 | 0.000 | 0.000 | 0.022 | 0.000 |
| g__Syntrophococcus | 0.000 | 0.000 | 0.000 | 0.000 | 0.021 | 0.000 |
| g__Gordonia | 0.000 | 0.000 | 0.000 | 0.000 | 0.018 | 0.000 |
| g__Candidatus_Udaeobacter | 0.000 | 0.000 | 0.000 | 0.000 | 0.018 | 0.000 |
| g__Zoogloea | 0.000 | 0.000 | 0.007 | 0.000 | 0.017 | 0.000 |
| g__Candidatus_Saccharimonas | 0.000 | 0.000 | 0.000 | 0.000 | 0.015 | 0.000 |
| g__Shuttleworthia | 0.000 | 0.000 | 0.000 | 0.000 | 0.014 | 0.000 |
| g__Subgroup_2_unclassified | 0.000 | 0.000 | 0.000 | 0.000 | 0.014 | 0.000 |
| g__Vibrio | 0.000 | 0.009 | 0.000 | 0.000 | 0.014 | 0.000 |
| g__JG30-KF-CM45_unclassified | 0.000 | 0.000 | 0.000 | 0.000 | 0.012 | 0.000 |
| g__Pseudactinotalea | 0.000 | 0.000 | 0.000 | 0.000 | 0.011 | 0.000 |
| g__SM1A02 | 0.000 | 0.000 | 0.000 | 0.000 | 0.011 | 0.000 |
| g__Prevotella_7 | 0.010 | 0.000 | 0.000 | 0.051 | 0.009 | 0.000 |
| g__Erysipelotrichaceae_UCG-009 | 0.000 | 0.000 | 0.000 | 0.000 | 0.008 | 0.000 |
| g__Chitinophagaceae_unclassified | 0.000 | 0.000 | 0.003 | 0.000 | 0.008 | 0.000 |
| g__Xanthobacteraceae_unclassified | 0.000 | 0.000 | 0.000 | 0.000 | 0.007 | 0.000 |
| g__Advenella | 0.000 | 0.000 | 0.000 | 0.000 | 0.007 | 0.000 |
| g__Succinivibrio | 0.000 | 0.000 | 0.000 | 0.000 | 0.007 | 0.000 |
| g__SM2D12_unclassified | 0.000 | 0.000 | 0.000 | 0.000 | 0.007 | 0.000 |
| g__Acidobacteriales_unclassified | 0.000 | 0.000 | 0.000 | 0.000 | 0.007 | 0.000 |
| g__Firmicutes_unclassified | 0.001 | 0.000 | 0.000 | 0.000 | 0.007 | 0.000 |
| g__Candidatus_Berkiella | 0.000 | 0.000 | 0.000 | 0.019 | 0.006 | 0.000 |
| g__WCHB1-41_unclassified | 0.000 | 0.000 | 0.000 | 0.000 | 0.006 | 0.000 |
| g__Duganella | 0.000 | 0.000 | 0.000 | 0.000 | 0.005 | 0.000 |
| g__Aquincola | 0.000 | 0.000 | 0.000 | 0.000 | 0.005 | 0.000 |
| g__Faecalibacterium | 0.000 | 0.000 | 0.000 | 0.000 | 0.005 | 0.000 |
| g__Halotalea | 0.000 | 0.000 | 0.000 | 0.000 | 0.005 | 0.000 |
| g__Christensenellaceae_R-7_group | 0.000 | 0.005 | 0.000 | 0.000 | 0.005 | 0.000 |
| g__Desulfovibrio | 0.000 | 0.002 | 0.000 | 0.000 | 0.005 | 0.000 |
| g__Acidothermus | 0.000 | 0.000 | 0.000 | 0.000 | 0.005 | 0.000 |
| g__Rahnella | 0.000 | 0.000 | 0.000 | 0.026 | 0.005 | 0.000 |
| g__Alphaproteobacteria_unclassified | 0.000 | 0.030 | 0.000 | 0.001 | 0.005 | 0.000 |
| g__Frigoribacterium | 0.000 | 0.000 | 0.000 | 0.000 | 0.005 | 0.000 |
| g__Ruminococcus_2 | 0.000 | 0.000 | 0.000 | 0.000 | 0.004 | 0.000 |
| g__Lachnospiraceae_NK4A136_group | 0.000 | 0.000 | 0.000 | 0.000 | 0.004 | 0.000 |
| g__Solitalea | 0.000 | 0.013 | 0.000 | 0.000 | 0.004 | 0.000 |
| g__Acidaminococcus | 0.000 | 0.000 | 0.000 | 0.000 | 0.004 | 0.000 |
| g__Roseburia | 0.000 | 0.000 | 0.000 | 0.000 | 0.004 | 0.000 |
| g__Elizabethkingia | 0.000 | 0.000 | 0.000 | 0.000 | 0.004 | 0.000 |
| g__Rhizobacter | 0.000 | 0.000 | 0.000 | 0.000 | 0.004 | 0.000 |
| g__Rhodospirillales_unclassified | 0.000 | 0.000 | 0.000 | 0.000 | 0.004 | 0.000 |
| g__Rokubacteriales_unclassified | 0.000 | 0.000 | 0.000 | 0.000 | 0.004 | 0.000 |
| g__Bilophila | 0.000 | 0.000 | 0.000 | 0.000 | 0.004 | 0.000 |
| g__Eubacterium]_nodatum_group | 0.000 | 0.000 | 0.000 | 0.000 | 0.003 | 0.000 |
| g__Aquimonas | 0.000 | 0.000 | 0.000 | 0.000 | 0.003 | 0.000 |
| g__Terrimonas | 0.000 | 0.000 | 0.000 | 0.000 | 0.003 | 0.000 |
| g__Alloprevotella | 0.000 | 0.000 | 0.000 | 0.000 | 0.003 | 0.000 |
| g__Leucobacter | 0.000 | 0.000 | 0.000 | 0.000 | 0.003 | 0.000 |
| g__Solobacterium | 0.034 | 0.000 | 0.000 | 0.000 | 0.003 | 0.000 |
| g__AD3_unclassified | 0.000 | 0.000 | 0.000 | 0.000 | 0.003 | 0.000 |
| g__Pleomorphomonadaceae_unclassified | 0.000 | 0.000 | 0.000 | 0.000 | 0.003 | 0.000 |
| g__Rikenellaceae_RC9_gut_group | 0.000 | 0.000 | 0.000 | 0.064 | 0.003 | 0.000 |
| g__Moryella | 0.000 | 0.000 | 0.000 | 0.000 | 0.003 | 0.000 |
| g__Parasutterella | 0.000 | 0.000 | 0.000 | 0.000 | 0.002 | 0.000 |
| g__Caulobacter | 0.000 | 0.000 | 0.000 | 0.000 | 0.002 | 0.000 |
| g__Tyzzerella | 0.000 | 0.000 | 0.000 | 0.000 | 0.002 | 0.000 |
| g__Lachnospiraceae_XPB1014_group | 0.000 | 0.000 | 0.000 | 0.000 | 0.002 | 0.000 |
| g__Christensenellaceae_unclassified | 0.003 | 0.005 | 0.000 | 0.000 | 0.002 | 0.000 |
| g__Burkholderiales_unclassified | 0.000 | 0.008 | 0.000 | 0.000 | 0.002 | 0.000 |
| g__Pyramidobacter | 0.000 | 0.000 | 0.000 | 0.000 | 0.002 | 0.000 |
| g__Gemmataceae_unclassified | 0.000 | 0.000 | 0.000 | 0.000 | 0.002 | 0.000 |
| g__Bryobacter | 0.000 | 0.000 | 0.000 | 0.000 | 0.002 | 0.000 |
| g__Candidatus_Solibacter | 0.000 | 0.000 | 0.000 | 0.000 | 0.002 | 0.000 |
| g__Candidatus_Methylopumilus | 0.000 | 0.000 | 0.000 | 0.000 | 0.002 | 0.000 |
| g__Phascolarctobacterium | 0.000 | 0.002 | 0.000 | 0.012 | 0.002 | 0.000 |
| g__Ruminococcus_1 | 0.000 | 0.000 | 0.000 | 0.000 | 0.002 | 0.000 |
| g__Reyranella | 0.000 | 0.000 | 0.000 | 0.000 | 0.002 | 0.000 |
| g__Eubacterium]_xylanophilum_group | 0.000 | 0.000 | 0.000 | 0.000 | 0.002 | 0.000 |
| g__Ruminiclostridium | 0.000 | 0.000 | 0.000 | 0.000 | 0.002 | 0.000 |
| g__JGI_0000069-P22_unclassified | 0.000 | 0.000 | 0.000 | 0.000 | 0.002 | 0.000 |
| g__Butyrivibrio | 0.000 | 0.000 | 0.000 | 0.000 | 0.002 | 0.000 |
| g__Desulfarculaceae_unclassified | 0.000 | 0.000 | 0.000 | 0.000 | 0.002 | 0.000 |
| g__Saprospiraceae_unclassified | 0.000 | 0.000 | 0.000 | 0.000 | 0.002 | 0.000 |
| g__Subgroup_5_unclassified | 0.000 | 0.000 | 0.000 | 0.000 | 0.002 | 0.000 |
| g__Vermiphilaceae_unclassified | 0.000 | 0.000 | 0.000 | 0.000 | 0.002 | 0.000 |
| g__Oscillibacter | 0.000 | 0.000 | 0.000 | 0.000 | 0.001 | 0.000 |
| g__Prevotella_1 | 0.000 | 0.000 | 0.000 | 0.000 | 0.001 | 0.000 |
| g__Dorea | 0.000 | 0.000 | 0.000 | 0.131 | 0.001 | 0.000 |
| g__Clostridiales_unclassified | 0.000 | 0.000 | 0.000 | 0.000 | 0.001 | 0.000 |
| g__Gastranaerophilales_unclassified | 0.000 | 0.000 | 0.000 | 0.000 | 0.001 | 0.000 |
| g__Coriobacteriaceae_unclassified | 0.000 | 0.000 | 0.000 | 0.000 | 0.001 | 0.000 |
| g__Coriobacteriales_unclassified | 0.000 | 0.000 | 0.000 | 0.000 | 0.001 | 0.000 |
| g__Erysipelotrichaceae_UCG-002 | 0.000 | 0.000 | 0.000 | 0.000 | 0.001 | 0.000 |
| g__JG30-KF-AS9_unclassified | 0.000 | 0.000 | 0.000 | 0.000 | 0.001 | 0.000 |
| g__Microlunatus | 0.000 | 0.000 | 0.000 | 0.000 | 0.001 | 0.000 |
| g__Arenimonas | 0.000 | 0.000 | 0.002 | 0.000 | 0.001 | 0.000 |
| g__Sphingobium | 0.000 | 0.028 | 0.000 | 0.000 | 0.001 | 0.000 |
| g__KD4-96_unclassified | 0.002 | 0.000 | 0.000 | 0.000 | 0.001 | 0.000 |
| g__RB41 | 0.000 | 0.000 | 0.000 | 0.000 | 0.001 | 0.000 |
| g__Family_XIII_UCG-001 | 0.000 | 0.000 | 0.000 | 0.000 | 0.001 | 0.000 |
| g__Gitt-GS-136_unclassified | 0.000 | 0.000 | 0.000 | 0.000 | 0.001 | 0.000 |
| g__Atopobiaceae_unclassified | 0.000 | 0.000 | 0.000 | 0.000 | 0.001 | 0.000 |
| g__Candidatus_Xiphinematobacter | 0.000 | 0.000 | 0.000 | 0.000 | 0.001 | 0.000 |
| g__Chthonomonas | 0.000 | 0.000 | 0.000 | 0.000 | 0.001 | 0.000 |
| g__Paracaedibacteraceae_unclassified | 0.000 | 0.000 | 0.000 | 0.000 | 0.001 | 0.000 |
| g__Paraprevotella | 0.000 | 0.000 | 0.000 | 0.000 | 0.001 | 0.000 |
| g__Curtobacterium | 0.000 | 0.071 | 0.000 | 0.145 | 0.000 | 0.000 |
| g__Saccharimonadaceae_unclassified | 0.000 | 0.026 | 0.000 | 0.140 | 0.000 | 0.000 |
| g__Enterobacteriaceae_unclassified | 0.000 | 0.028 | 0.000 | 0.123 | 0.000 | 0.000 |
| g__Deltaproteobacteria_unclassified | 0.000 | 0.005 | 0.000 | 0.101 | 0.000 | 0.000 |
| g__Glutamicibacter | 0.000 | 0.000 | 0.000 | 0.083 | 0.000 | 0.000 |
| g__Pantoea | 0.000 | 0.423 | 0.000 | 0.082 | 0.000 | 0.000 |
| g__Romboutsia | 0.000 | 0.000 | 0.000 | 0.081 | 0.000 | 0.000 |
| g__Dietzia | 0.000 | 0.000 | 0.000 | 0.079 | 0.000 | 0.000 |
| g__Hymenobacter | 0.000 | 0.076 | 0.000 | 0.064 | 0.000 | 0.000 |
| g__Labedella | 0.004 | 0.291 | 0.000 | 0.057 | 0.000 | 0.000 |
| g__Megamonas | 0.000 | 0.001 | 0.000 | 0.037 | 0.000 | 0.000 |
| g__Erwinia | 0.000 | 0.000 | 0.000 | 0.036 | 0.000 | 0.000 |
| g__Eubacterium]_hallii_group | 0.000 | 0.006 | 0.000 | 0.033 | 0.000 | 0.000 |
| g__Deinococcus | 0.000 | 0.037 | 0.000 | 0.031 | 0.000 | 0.000 |
| g__Mastigocladopsis_PCC-10914 | 0.000 | 0.000 | 0.000 | 0.026 | 0.000 | 0.000 |
| g__Halobacillus | 0.000 | 0.000 | 0.000 | 0.025 | 0.000 | 0.000 |
| g__Acidiphilium | 0.000 | 0.000 | 0.000 | 0.021 | 0.000 | 0.000 |
| g__Ornithinimicrobium | 0.000 | 0.000 | 0.000 | 0.017 | 0.000 | 0.000 |
| g__SJA-28_unclassified | 0.000 | 0.000 | 0.000 | 0.016 | 0.000 | 0.000 |
| g__Arsenicitalea | 0.000 | 0.000 | 0.000 | 0.015 | 0.000 | 0.000 |
| g__Ensifer | 0.000 | 0.021 | 0.000 | 0.014 | 0.000 | 0.000 |
| g__1174-901-12 | 0.151 | 0.003 | 0.000 | 0.011 | 0.000 | 0.000 |
| g__Facklamia | 0.000 | 0.000 | 0.000 | 0.011 | 0.000 | 0.000 |
| g__Candidatus_Jidaibacter | 0.000 | 0.000 | 0.000 | 0.009 | 0.000 | 0.000 |
| g__Prevotella_9 | 0.000 | 0.000 | 0.000 | 0.009 | 0.000 | 0.000 |
| g__Anaerobacillus | 0.000 | 0.000 | 0.000 | 0.009 | 0.000 | 0.000 |
| g__Turicibacter | 0.000 | 0.000 | 0.000 | 0.009 | 0.000 | 0.000 |
| g__Truepera | 0.000 | 0.015 | 0.000 | 0.007 | 0.000 | 0.000 |
| g__Pelagibacterium | 0.000 | 0.000 | 0.000 | 0.005 | 0.000 | 0.000 |
| g__Alkanindiges | 0.000 | 0.000 | 0.000 | 0.004 | 0.000 | 0.000 |
| g__Serratia | 0.000 | 0.000 | 0.000 | 0.003 | 0.000 | 0.000 |
| g__Coriobacteriaceae_UCG-002 | 0.000 | 0.000 | 0.000 | 0.003 | 0.000 | 0.000 |
| g__Sporosarcina | 0.000 | 0.000 | 0.000 | 0.002 | 0.000 | 0.000 |
| g__Arcticibacter | 0.000 | 0.000 | 0.000 | 0.002 | 0.000 | 0.000 |
| g__Mesorhizobium | 0.000 | 0.000 | 0.000 | 0.002 | 0.000 | 0.000 |
| g__Xylophilus | 0.000 | 0.000 | 0.000 | 0.002 | 0.000 | 0.000 |
| g__Neisseriaceae_unclassified | 0.000 | 0.000 | 0.000 | 0.002 | 0.000 | 0.000 |
| g__Clostridium_sensu_stricto_8 | 0.000 | 0.000 | 0.043 | 0.000 | 0.000 | 0.000 |
| g__Limnobacter | 0.000 | 0.000 | 0.022 | 0.000 | 0.000 | 0.000 |
| g__Tepidimonas | 0.000 | 0.000 | 0.019 | 0.000 | 0.000 | 0.000 |
| g__PMMR1 | 0.000 | 0.000 | 0.011 | 0.000 | 0.000 | 0.000 |
| g__Actinoplanes | 0.000 | 0.000 | 0.008 | 0.000 | 0.000 | 0.000 |
| g__Flavobacterium | 0.000 | 0.003 | 0.007 | 0.000 | 0.000 | 0.000 |
| g__Brevibacillus | 0.000 | 0.000 | 0.006 | 0.000 | 0.000 | 0.000 |
| g__Planococcaceae_unclassified | 0.000 | 0.000 | 0.003 | 0.000 | 0.000 | 0.000 |
| g__MBA03_unclassified | 0.000 | 0.000 | 0.003 | 0.000 | 0.000 | 0.000 |
| g__Methylophilaceae_unclassified | 0.000 | 0.001 | 0.003 | 0.000 | 0.000 | 0.000 |
| g__Nordella | 0.000 | 0.000 | 0.002 | 0.000 | 0.000 | 0.000 |
| g__Cloacibacillus | 0.000 | 0.000 | 0.002 | 0.000 | 0.000 | 0.000 |
| g__Cryomorphaceae_unclassified | 0.000 | 0.000 | 0.002 | 0.000 | 0.000 | 0.000 |
| g__Undibacterium | 0.000 | 0.000 | 0.002 | 0.000 | 0.000 | 0.000 |
| g__Methylophilus | 0.000 | 0.003 | 0.002 | 0.000 | 0.000 | 0.000 |
| g__Desulfosporosinus | 0.000 | 0.000 | 0.002 | 0.000 | 0.000 | 0.000 |
| g__DTU014_unclassified | 0.000 | 0.000 | 0.001 | 0.000 | 0.000 | 0.000 |
| g__Anaerolineaceae_unclassified | 0.000 | 0.000 | 0.001 | 0.000 | 0.000 | 0.000 |
| g__Proteobacteria_unclassified | 0.000 | 0.000 | 0.001 | 0.000 | 0.000 | 0.000 |
| g__WS6_(Dojkabacteria)_unclassified | 0.000 | 0.000 | 0.001 | 0.000 | 0.000 | 0.000 |
| g__Dermacoccus | 0.000 | 0.018 | 0.001 | 0.000 | 0.000 | 0.000 |
| g__Aminicenantales_unclassified | 0.000 | 0.000 | 0.001 | 0.000 | 0.000 | 0.000 |
| g__Prolixibacteraceae_unclassified | 0.000 | 0.000 | 0.001 | 0.000 | 0.000 | 0.000 |
| g__Spirosoma | 0.000 | 0.383 | 0.000 | 0.000 | 0.000 | 0.000 |
| g__Cyanobacteria_unclassified | 0.000 | 0.101 | 0.000 | 0.000 | 0.000 | 0.000 |
| g__Rhodopseudomonas | 0.023 | 0.068 | 0.000 | 0.000 | 0.000 | 0.000 |
| g__Leifsonia | 0.000 | 0.054 | 0.000 | 0.000 | 0.000 | 0.000 |
| g__Leptolyngbya_PCC-6306 | 0.000 | 0.038 | 0.000 | 0.000 | 0.000 | 0.000 |
| g__Craurococcus | 0.000 | 0.034 | 0.000 | 0.000 | 0.000 | 0.000 |
| g__Sediminibacterium | 0.000 | 0.028 | 0.000 | 0.000 | 0.000 | 0.000 |
| g__Leptotrichiaceae_unclassified | 0.000 | 0.025 | 0.000 | 0.000 | 0.000 | 0.000 |
| g__Prevotellaceae_UCG-003 | 0.000 | 0.024 | 0.000 | 0.000 | 0.000 | 0.000 |
| g__AB1_unclassified | 0.000 | 0.023 | 0.000 | 0.000 | 0.000 | 0.000 |
| g__Larkinella | 0.000 | 0.019 | 0.000 | 0.000 | 0.000 | 0.000 |
| g__Bacillaceae_unclassified | 0.000 | 0.014 | 0.000 | 0.000 | 0.000 | 0.000 |
| g__Johnsonella | 0.000 | 0.012 | 0.000 | 0.000 | 0.000 | 0.000 |
| g__Bacteroidetes_unclassified | 0.000 | 0.011 | 0.000 | 0.000 | 0.000 | 0.000 |
| g__Chryseomicrobium | 0.000 | 0.010 | 0.000 | 0.000 | 0.000 | 0.000 |
| g__Raoultella | 0.000 | 0.010 | 0.000 | 0.000 | 0.000 | 0.000 |
| g__Methylobacillus | 0.000 | 0.009 | 0.000 | 0.000 | 0.000 | 0.000 |
| g__Patulibacter | 0.000 | 0.009 | 0.000 | 0.000 | 0.000 | 0.000 |
| g__Chroococcidiopsis_PCC_7203 | 0.000 | 0.008 | 0.000 | 0.000 | 0.000 | 0.000 |
| g__Quadrisphaera | 0.000 | 0.007 | 0.000 | 0.000 | 0.000 | 0.000 |
| g__Gemmata | 0.000 | 0.007 | 0.000 | 0.000 | 0.000 | 0.000 |
| g__NB1-j_unclassified | 0.000 | 0.007 | 0.000 | 0.000 | 0.000 | 0.000 |
| g__Psychroglaciecola | 0.000 | 0.006 | 0.000 | 0.000 | 0.000 | 0.000 |
| g__Adhaeribacter | 0.000 | 0.006 | 0.000 | 0.000 | 0.000 | 0.000 |
| g__Rickettsiaceae_unclassified | 0.000 | 0.005 | 0.000 | 0.000 | 0.000 | 0.000 |
| g__Rhodocytophaga | 0.000 | 0.005 | 0.000 | 0.000 | 0.000 | 0.000 |
| g__Rhodovarius | 0.000 | 0.005 | 0.000 | 0.000 | 0.000 | 0.000 |
| g__Legionella | 0.000 | 0.005 | 0.000 | 0.000 | 0.000 | 0.000 |
| g__CENA359 | 0.000 | 0.004 | 0.000 | 0.000 | 0.000 | 0.000 |
| g__Eubacterium]_ruminantium_group | 0.001 | 0.004 | 0.000 | 0.000 | 0.000 | 0.000 |
| g__Sphingoaurantiacus | 0.000 | 0.004 | 0.000 | 0.000 | 0.000 | 0.000 |
| g__S085_unclassified | 0.000 | 0.004 | 0.000 | 0.000 | 0.000 | 0.000 |
| g__Rubritepida | 0.000 | 0.004 | 0.000 | 0.000 | 0.000 | 0.000 |
| g__Armatimonadales_unclassified | 0.000 | 0.003 | 0.000 | 0.000 | 0.000 | 0.000 |
| g__Thermicanus | 0.000 | 0.003 | 0.000 | 0.000 | 0.000 | 0.000 |
| g__Croceifilum | 0.000 | 0.003 | 0.000 | 0.000 | 0.000 | 0.000 |
| g__Calothrix_PCC-6303 | 0.000 | 0.003 | 0.000 | 0.000 | 0.000 | 0.000 |
| g__Cnuella | 0.000 | 0.003 | 0.000 | 0.000 | 0.000 | 0.000 |
| g__Spirosomaceae_unclassified | 0.000 | 0.003 | 0.000 | 0.000 | 0.000 | 0.000 |
| g__Coprococcus_2 | 0.002 | 0.003 | 0.000 | 0.000 | 0.000 | 0.000 |
| g__Papillibacter | 0.000 | 0.003 | 0.000 | 0.000 | 0.000 | 0.000 |
| g__MN_122.2a | 0.000 | 0.002 | 0.000 | 0.000 | 0.000 | 0.000 |
| g__Dyadobacter | 0.000 | 0.002 | 0.000 | 0.000 | 0.000 | 0.000 |
| g__Ruminiclostridium_6 | 0.000 | 0.002 | 0.000 | 0.000 | 0.000 | 0.000 |
| g__Clade_III_unclassified | 0.000 | 0.002 | 0.000 | 0.000 | 0.000 | 0.000 |
| g__Ruminococcaceae_UCG-005 | 0.000 | 0.002 | 0.000 | 0.000 | 0.000 | 0.000 |
| g__Ruminococcaceae_UCG-010 | 0.000 | 0.002 | 0.000 | 0.000 | 0.000 | 0.000 |
| g__Clostridium | 0.000 | 0.002 | 0.000 | 0.000 | 0.000 | 0.000 |
| g__TRA3-20_unclassified | 0.000 | 0.002 | 0.000 | 0.000 | 0.000 | 0.000 |
| g__Tyzzerella_3 | 0.000 | 0.001 | 0.000 | 0.000 | 0.000 | 0.000 |
| g__Ruminiclostridium_1 | 0.000 | 0.001 | 0.000 | 0.000 | 0.000 | 0.000 |
| g__Chromobacterium | 0.000 | 0.001 | 0.000 | 0.000 | 0.000 | 0.000 |
| g__Gibbsiella | 0.000 | 0.001 | 0.000 | 0.000 | 0.000 | 0.000 |
| g__Psychrobacter | 0.159 | 0.000 | 0.000 | 0.000 | 0.000 | 0.000 |
| g__Pseudarthrobacter | 0.125 | 0.000 | 0.000 | 0.000 | 0.000 | 0.000 |
| g__Paenisporosarcina | 0.010 | 0.000 | 0.000 | 0.000 | 0.000 | 0.000 |
| g__Virgibacillus | 0.002 | 0.000 | 0.000 | 0.000 | 0.000 | 0.000 |
| g__Phreatobacter | 0.002 | 0.000 | 0.000 | 0.000 | 0.000 | 0.000 |
| g__Lysinibacillus | 0.001 | 0.000 | 0.000 | 0.000 | 0.000 | 0.000 |

Table S5 Bacteria had important influence on volatile components

| Bacterium | VIP value |
| --- | --- |
| g__Coprococcus_2 | 1.53194 |
| g__Mitochondria_unclassified | 1.41582 |
| g__Microbacteriaceae_unclassified | 1.33126 |
| g__Christensenellaceae_unclassified | 1.31602 |
| g__Bifidobacterium | 1.31481 |
| g__Weissella | 1.31346 |
| g__1174-901-12 | 1.30064 |
| g__0319-6G20_unclassified | 1.30017 |
| g__Eubacterium]_ruminantium_group | 1.29866 |
| g__Rhodopseudomonas | 1.26986 |
| g__Solobacterium | 1.25229 |
| g__Herbaspirillum | 1.13615 |
| g__Rubellimicrobium | 1.13259 |
| g__Candidatus_Berkiella | 1.12091 |
| g__Bacillus | 1.09912 |
| g__Zoogloea | 1.07451 |
| g__Chitinophagaceae_unclassified | 1.07344 |
| g__Aquabacterium | 1.07221 |
| g__Rahnella | 1.06775 |
| g__Neisseria | 1.05544 |
| g__Fretibacterium | 1.05267 |
| g__Thermomonas | 1.05186 |
| g__Proteiniphilum | 1.05033 |
| g__Prevotella | 1.04731 |
| g__Delftia | 1.04658 |
| g__Akkermansia | 1.04508 |
| g__Candidatus_Bacilloplasma | 1.04231 |
| g__Roseomonas | 1.04142 |
| g__Burkholderia-Caballeronia-Paraburkholderia | 1.03885 |
| g__Dechloromonas | 1.03437 |
| g__Ralstonia | 1.03436 |
| g__Haemophilus | 1.03431 |
| g__Paracoccus | 1.03413 |
| g__Kocuria | 1.03223 |
| g__Streptococcus | 1.03221 |
| g__Enhydrobacter | 1.03179 |
| g__Escherichia-Shigella | 1.03164 |
| g__Luteimonas | 1.03141 |
| g__Corynebacterium | 1.03102 |
| g__Lautropia | 1.03088 |
| g__Actinobacteria_unclassified | 1.0301 |
| g__Arthrobacter | 1.03002 |
| g__Renibacterium | 1.0299 |
| g__Eubacterium]_coprostanoligenes_group | 1.0299 |
| g__Ruminococcaceae_UCG-014 | 1.02987 |
| g__Ruminococcus]_gnavus_group | 1.02986 |
| g__Ruminococcaceae_NK4A214_group | 1.02975 |
| g__Sphingopyxis | 1.02953 |
| g__Hydrogenophaga | 1.02946 |
| g__Acidibacter | 1.02943 |
| g__Parabacteroides | 1.02934 |
| g__Actinomyces | 1.0293 |
| g__Pseudoxanthomonas | 1.02929 |
| g__Helicobacter | 1.02926 |
| g__Corynebacterium_1 | 1.02922 |
| g__Veillonellaceae_unclassified | 1.02906 |
| g__Shinella | 1.02905 |
| g__Novosphingobium | 1.02901 |
| g__Cloacibacterium | 1.02898 |
| g__Enterococcus | 1.02895 |
| g__Fusobacterium | 1.02891 |
| g__Sphingobacterium | 1.0289 |
| g__Dialister | 1.02881 |
| g__Paenibacillus | 1.02879 |
| g__Schlegelella | 1.02879 |
| g__Bergeyella | 1.02877 |
| g__Lawsonella | 1.02862 |
| g__Brevundimonas | 1.02842 |
| g__Cellulomonas | 1.02842 |
| g__Janibacter | 1.02827 |
| g__Micrococcus | 1.02817 |
| g__Rothia | 1.02805 |
| g__Cutibacterium | 1.02762 |
| g__Blautia | 1.02741 |
| g__Gardnerella | 1.02738 |
| g__Stenotrophomonas | 1.02719 |
| g__unclassified | 1.02698 |
| g__Weeksellaceae_unclassified | 1.0269 |
| g__Staphylococcus | 1.02683 |
| g__Bacteroides | 1.02661 |
| g__Porphyrobacter | 1.02638 |
| g__Geodermatophilus | 1.02608 |
| g__Aeromonas | 1.02519 |
| g__Klebsiella | 1.02477 |
| g__Lactobacillus | 1.02445 |
| g__Oceanobacillus | 1.02437 |
| g__Pseudomonas | 1.02436 |
| g__Brachybacterium | 1.02416 |
| g__Beijerinckiaceae_unclassified | 1.02402 |
| g__Gemmatirosa | 1.0238 |
| g__Comamonas | 1.02332 |
| g__Variovorax | 1.02299 |
| g__Ruminococcus]_torques_group | 1.02214 |
| g__Pelomonas | 1.02199 |
| g__Pseudoramibacter | 1.02198 |
| g__Acinetobacter | 1.02144 |
| g__Bradyrhizobium | 1.02121 |
| g__Clavibacter | 1.02085 |
| g__Anaerococcus | 1.02003 |
| g__Exiguobacterium | 1.0177 |
| g__Devosia | 1.01612 |
| g__Gemmatimonas | 1.01179 |
| g__Campylobacter | 1.01161 |
| g__Solitalea | 1.01156 |
| g__Massilia | 1.0103 |
| g__Lachnospiraceae_unclassified | 1.01006 |
| g__Curvibacter | 1.00977 |
| g__Burkholderiales_unclassified | 1.00849 |
| g__Mucilaginibacter | 1.00637 |
| g__Gammaproteobacteria_unclassified | 1.00462 |
| g__Christensenellaceae_R-7_group | 1.00338 |
| g__Aliterella_CENA595 | 1.00233 |
| g__Kineococcus | 1.00204 |
| g__Rikenellaceae_RC9_gut_group | 1.00182 |
| g__Oxyphotobacteria_unclassified | 1.00172 |
| g__Bdellovibrio | 1.00026 |

Table S6 Correlation between volatile components and bacteria (*P* < 0.05)

| Volatile components | Bacteria | Correlation coefficient | Volatile components | Bacteria | Correlation coefficient | Volatile components | Bacteria | Correlation coefficient |
| --- | --- | --- | --- | --- | --- | --- | --- | --- |
| Octanal D | Oxyphotobacteria unclassified | -0.903 | (E,E)-2,4-Heptadienal | Paenibacillus | 0.894 | 2-Heptanone M | Bacteroides | 0.902 |
| (E)-2-Heptenal | Oxyphotobacteria unclassified | -0.911 | Octanal D | Paenibacillus | 0.941 | 2-Pentyl furan | Bacteroides | 0.817 |
| 3-Methylbutanal M | Oxyphotobacteria unclassified | -0.829 | Octanal M | Paenibacillus | 0.829 | Nonanal M | Novosphingobium | 0.877 |
| Hexanal T | Oxyphotobacteria unclassified | -0.945 | (E)-2-Heptenal | Paenibacillus | 0.821 | Nonanal D | Novosphingobium | 0.873 |
| Heptanal M | Oxyphotobacteria unclassified | -0.820 | Hexanal T | Paenibacillus | 0.916 | (E,E)-2,4-Heptadienal | Novosphingobium | 0.891 |
| Heptanal D | Oxyphotobacteria unclassified | -0.922 | Heptanal M | Paenibacillus | 0.876 | Octanal D | Novosphingobium | 0.936 |
| Trimethylpyrazine | Oxyphotobacteria unclassified | -0.814 | Heptanal D | Paenibacillus | 0.978 | Octanal M | Novosphingobium | 0.827 |
| Nonanal D | Brevundimonas | 0.875 | 3-Methyl-1-butanol | Paenibacillus | -0.821 | (E)-2-Heptenal | Novosphingobium | 0.813 |
| (E,E)-2,4-Heptadienal | Brevundimonas | 0.869 | 2-Heptanone M | Paenibacillus | 0.911 | Hexanal T | Novosphingobium | 0.911 |
| Octanal D | Brevundimonas | 0.885 | 2-Pentyl furan | Paenibacillus | 0.828 | Heptanal M | Novosphingobium | 0.873 |
| Octanal M | Brevundimonas | 0.931 | Nonanal M | Burkholderia | 0.849 | Heptanal D | Novosphingobium | 0.974 |
| (E)-2-Heptenal | Brevundimonas | 0.820 | Nonanal D | Burkholderia | 0.853 | 3-Methyl-1-butanol | Novosphingobium | -0.828 |
| Heptanal M | Brevundimonas | 0.908 | (E,E)-2,4-Heptadienal | Burkholderia | 0.926 | 2-Heptanone M | Novosphingobium | 0.912 |
| Heptanal D | Brevundimonas | 0.865 | Octanal D | Burkholderia | 0.938 | 2-Pentyl furan | Novosphingobium | 0.822 |
| Linalool M | Brevundimonas | 0.971 | Octanal M | Burkholderia | 0.856 | (E)-2-Octenal | Actinobacteria unclassified | 0.847 |
| 2-Heptanone M | Brevundimonas | -0.835 | (E)-2-Heptenal | Burkholderia | 0.825 | Nonanal M | Actinobacteria unclassified | 0.883 |
| Trimethylpyrazine | Brevundimonas | 0.913 | Hexanal T | Burkholderia | 0.895 | Nonanal D | Actinobacteria unclassified | 0.894 |
| 2-Acetylfuran | Brevundimonas | 0.814 | Heptanal M | Burkholderia | 0.886 | (E,E)-2,4-Heptadienal | Actinobacteria unclassified | 0.907 |
| Nonanal M | Lawsonella | 0.874 | Heptanal D | Burkholderia | 0.969 | Octanal D | Actinobacteria unclassified | 0.973 |
| Nonanal D | Lawsonella | 0.870 | 3-Methyl-1-butanol | Burkholderia | -0.817 | Octanal M | Actinobacteria unclassified | 0.836 |
| (E,E)-2,4-Heptadienal | Lawsonella | 0.885 | 2-Heptanone M | Burkholderia | 0.877 | (E)-2-Heptenal | Actinobacteria unclassified | 0.885 |
| Octanal D | Lawsonella | 0.935 | 2-Pentyl furan | Burkholderia | 0.844 | Hexanal T | Actinobacteria unclassified | 0.952 |
| Octanal M | Lawsonella | 0.817 | (E,E)-2,4-Heptadienal | Bacillus | 0.905 | Heptanal M | Actinobacteria unclassified | 0.881 |
| (E)-2-Heptenal | Lawsonella | 0.812 | Octanal D | Bacillus | 0.814 | Heptanal D | Actinobacteria unclassified | 0.994 |
| Hexanal T | Lawsonella | 0.914 | Octanal M | Bacillus | 0.835 | 2-Heptanone M | Actinobacteria unclassified | 0.87 |
| Heptanal M | Lawsonella | 0.864 | Heptanal M | Bacillus | 0.851 | Trimethylpyrazine | Actinobacteria unclassified | 0.835 |
| Heptanal D | Lawsonella | 0.974 | Heptanal D | Bacillus | 0.866 | 2-Pentyl furan | Actinobacteria unclassified | 0.862 |
| 3-Methyl-1-butanol | Lawsonella | -0.834 | 3-Methyl-1-butanol | Bacillus | -0.825 | Nonanal M | Klebsiella | 0.864 |
| 2-Heptanone M | Lawsonella | 0.908 | Nonanal M | Ralstonia | 0.846 | Nonanal D | Klebsiella | 0.861 |
| 2-Pentyl furan | Lawsonella | 0.816 | Nonanal D | Ralstonia | 0.840 | (E,E)-2,4-Heptadienal | Klebsiella | 0.875 |
| Nonanal M | Cutibacterium | 0.873 | (E,E)-2,4-Heptadienal | Ralstonia | 0.895 | Octanal D | Klebsiella | 0.937 |
| Nonanal D | Cutibacterium | 0.867 | Octanal D | Ralstonia | 0.917 | (E)-2-Heptenal | Klebsiella | 0.82 |
| (E,E)-2,4-Heptadienal | Cutibacterium | 0.885 | Octanal M | Ralstonia | 0.828 | Hexanal T | Klebsiella | 0.93 |
| Octanal D | Cutibacterium | 0.931 | Hexanal T | Ralstonia | 0.889 | Heptanal M | Klebsiella | 0.869 |
| Octanal M | Cutibacterium | 0.820 | Heptanal M | Ralstonia | 0.874 | Heptanal D | Klebsiella | 0.982 |
| Hexanal T | Cutibacterium | 0.910 | Heptanal D | Ralstonia | 0.964 | 3-Methyl-1-butanol | Klebsiella | -0.829 |
| Heptanal M | Cutibacterium | 0.870 | 3-Methyl-1-butanol | Ralstonia | -0.847 | 2-Heptanone M | Klebsiella | 0.898 |
| Heptanal D | Cutibacterium | 0.973 | 2-Heptanone M | Ralstonia | 0.900 | (E)-2-Octenal | Escherichia | 0.844 |
| 3-Methyl-1-butanol | Cutibacterium | -0.834 | Nonanal M | Lactobacillus | 0.873 | Nonanal M | Escherichia | 0.867 |
| 2-Heptanone M | Cutibacterium | 0.913 | Nonanal D | Lactobacillus | 0.866 | Nonanal D | Escherichia | 0.872 |
| 2-Pentyl furan | Cutibacterium | 0.815 | (E,E)-2,4-Heptadienal | Lactobacillus | 0.870 | (E,E)-2,4-Heptadienal | Escherichia | 0.874 |
| Nonanal M | Schlegelella | 0.874 | Octanal D | Lactobacillus | 0.927 | Octanal D | Escherichia | 0.957 |
| Nonanal D | Schlegelella | 0.869 | Hexanal T | Lactobacillus | 0.913 | (E)-2-Heptenal | Escherichia | 0.861 |
| (E,E)-2,4-Heptadienal | Schlegelella | 0.885 | Heptanal M | Lactobacillus | 0.858 | Hexanal T | Escherichia | 0.959 |
| Octanal D | Schlegelella | 0.931 | Heptanal D | Lactobacillus | 0.971 | Heptanal M | Escherichia | 0.864 |
| Octanal M | Schlegelella | 0.819 | 3-Methyl-1-butanol | Lactobacillus | -0.839 | Heptanal D | Escherichia | 0.993 |
| Hexanal T | Schlegelella | 0.908 | 2-Heptanone M | Lactobacillus | 0.915 | 2-Heptanone M | Escherichia | 0.871 |
| Heptanal M | Schlegelella | 0.864 | Nonanal M | Fusobacterium | 0.875 | 2-Pentyl furan | Escherichia | 0.825 |
| Heptanal D | Schlegelella | 0.971 | Nonanal D | Fusobacterium | 0.87 | Nonanal M | Enhydrobacter | 0.872 |
| 3-Methyl-1-butanol | Schlegelella | -0.835 | (E,E)-2,4-Heptadienal | Fusobacterium | 0.888 | Nonanal D | Enhydrobacter | 0.865 |
| 2-Heptanone M | Schlegelella | 0.912 | Octanal D | Fusobacterium | 0.932 | (E,E)-2,4-Heptadienal | Enhydrobacter | 0.894 |
| 2-Pentyl furan | Schlegelella | 0.814 | Octanal M | Fusobacterium | 0.823 | Octanal D | Enhydrobacter | 0.927 |
| Nonanal M | Streptococcus | 0.859 | Hexanal T | Fusobacterium | 0.908 | Octanal M | Enhydrobacter | 0.831 |
| Nonanal D | Streptococcus | 0.854 | Heptanal M | Fusobacterium | 0.868 | Hexanal T | Enhydrobacter | 0.896 |
| (E,E)-2,4-Heptadienal | Streptococcus | 0.895 | Heptanal D | Fusobacterium | 0.972 | Heptanal M | Enhydrobacter | 0.873 |
| Octanal D | Streptococcus | 0.924 | 3-Methyl-1-butanol | Fusobacterium | -0.833 | Heptanal D | Enhydrobacter | 0.967 |
| Octanal M | Streptococcus | 0.830 | 2-Heptanone M | Fusobacterium | 0.912 | 3-Methyl-1-butanol | Enhydrobacter | -0.834 |
| Hexanal T | Streptococcus | 0.896 | 2-Pentyl furan | Fusobacterium | 0.817 | 2-Heptanone M | Enhydrobacter | 0.916 |
| Heptanal M | Streptococcus | 0.876 | (E)-2-Octenal | Acinetobacter | 0.812 | 2-Pentyl furan | Enhydrobacter | 0.818 |
| Heptanal D | Streptococcus | 0.968 | Nonanal M | Acinetobacter | 0.9 | Ethyl benzoate | Mitochondria unclassified | 0.837 |
| 3-Methyl-1-butanol | Streptococcus | -0.839 | Nonanal D | Acinetobacter | 0.892 | (E)-2-Octenal | Mitochondria unclassified | -0.833 |
| 2-Heptanone M | Streptococcus | 0.908 | (E,E)-2,4-Heptadienal | Acinetobacter | 0.886 | Hexanal T | Mitochondria unclassified | -0.815 |
| 2-Pentyl furan | Streptococcus | 0.815 | Octanal D | Acinetobacter | 0.938 | Linalool M | Mitochondria unclassified | 0.813 |
| (E)-2-Octenal | Staphylococcus | 0.817 | Octanal M | Acinetobacter | 0.832 | Linalool D | Mitochondria unclassified | 0.83 |
| Nonanal M | Staphylococcus | 0.879 | (E)-2-Heptenal | Acinetobacter | 0.815 | 2-Phenylethanol D | Mitochondria unclassified | 0.867 |
| Nonanal D | Staphylococcus | 0.878 | Hexanal T | Acinetobacter | 0.913 | 2-Phenylethanol M | Mitochondria unclassified | 0.875 |
| (E,E)-2,4-Heptadienal | Staphylococcus | 0.890 | Heptanal M | Acinetobacter | 0.884 | 2-Acetylfuran | Mitochondria unclassified | -0.89 |
| Octanal D | Staphylococcus | 0.948 | Heptanal D | Acinetobacter | 0.975 | Limonene | Mitochondria unclassified | 0.984 |
| Octanal M | Staphylococcus | 0.823 | 2-Heptanone M | Acinetobacter | 0.933 | Methyl salicylate | Microbacteriaceae unclassified | 1 |
| (E)-2-Heptenal | Staphylococcus | 0.836 | 2-Pentyl furan | Acinetobacter | 0.831 | Ethyl benzoate | Microbacteriaceae unclassified | 0.93 |
| Hexanal T | Staphylococcus | 0.932 | Nonanal M | Enterococcus | 0.875 | Pentanal D | Microbacteriaceae unclassified | 0.96 |
| Heptanal M | Staphylococcus | 0.878 | Nonanal D | Enterococcus | 0.87 | Linalool M | Microbacteriaceae unclassified | 0.987 |
| Heptanal D | Staphylococcus | 0.985 | (E,E)-2,4-Heptadienal | Enterococcus | 0.889 | Linalool D | Microbacteriaceae unclassified | 0.992 |
| 3-Methyl-1-butanol | Staphylococcus | -0.814 | Octanal D | Enterococcus | 0.933 | Linalool oxide M | Microbacteriaceae unclassified | 0.983 |
| 2-Heptanone M | Staphylococcus | 0.902 | Octanal M | Enterococcus | 0.825 | Linalool oxide D | Microbacteriaceae unclassified | 0.953 |
| 2-Pentyl furan | Staphylococcus | 0.831 | Hexanal T | Enterococcus | 0.909 | 2-Phenylethanol D | Microbacteriaceae unclassified | 0.978 |
| Nonanal M | Corynebacterium-1 | 0.878 | Heptanal M | Enterococcus | 0.871 | 2-Phenylethanol M | Microbacteriaceae unclassified | 0.94 |
| Nonanal D | Corynebacterium-1 | 0.876 | Heptanal D | Enterococcus | 0.973 | 1-Propanol D | Microbacteriaceae unclassified | -0.986 |
| (E,E)-2,4-Heptadienal | Corynebacterium-1 | 0.894 | 3-Methyl-1-butanol | Enterococcus | -0.831 | 2-6-Dimethylpyrazine | Microbacteriaceae unclassified | 0.897 |
| Octanal D | Corynebacterium-1 | 0.943 | 2-Heptanone M | Enterococcus | 0.913 | Styrene | Microbacteriaceae unclassified | -0.858 |
| Octanal M | Corynebacterium-1 | 0.828 | 2-Pentyl furan | Enterococcus | 0.819 | 3-Methyl-1-butanol | Bacteroides | -0.828 |
| (E)-2-Heptenal | Corynebacterium-1 | 0.827 | Nonanal M | Bacteroides | 0.87 | Nonanal M | Paenibacillus | 0.880 |
| Hexanal T | Corynebacterium-1 | 0.921 | Nonanal D | Bacteroides | 0.867 | Nonanal D | Paenibacillus | 0.877 |
| Heptanal M | Corynebacterium-1 | 0.874 | (E,E)-2,4-Heptadienal | Bacteroides | 0.883 | Heptanal M | Bacteroides | 0.871 |
| Heptanal D | Corynebacterium-1 | 0.980 | Octanal D | Bacteroides | 0.939 | Heptanal D | Bacteroides | 0.981 |
| 3-Methyl-1-butanol | Corynebacterium-1 | -0.821 | Octanal M | Bacteroides | 0.815 | 2-Pentyl furan | Corynebacterium-1 | 0.829 |
| 2-Heptanone M | Corynebacterium-1 | 0.905 | (E)-2-Heptenal | Bacteroides | 0.821 | Hexanal T | Bacteroides | 0.925 |


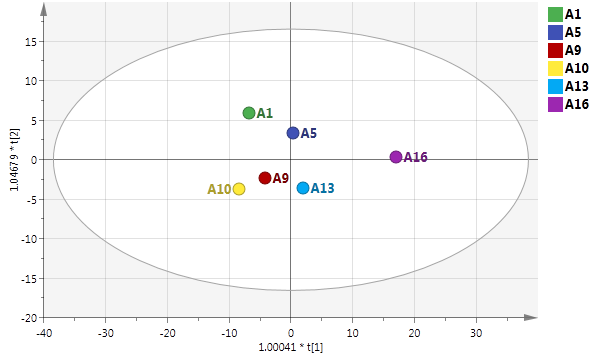


Figure S1 Score Chart of O2PLS


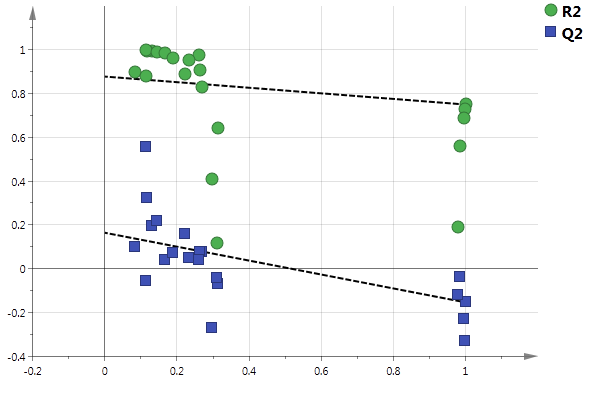


Figure S2 Cross validation of O2PLS
